# Unraveling Endocannabinoid Signaling Pathways in Cisplatin‐Induced Ototoxicity

**DOI:** 10.1096/fj.202502888RR

**Published:** 2026-02-17

**Authors:** Sakthimala Palaniappan, Annamaria Tisi, Camilla Di Meo, Cristina Urbano, Georgina E. Fenton, Francesco Della Valle, Federico Fanti, Dario Compagnone, Marc Nazarè, Huib Versnel, Andrea Aramini, Marcello Allegretti, Mauro Maccarrone

**Affiliations:** ^1^ Department of Biotechnological and Applied Clinical Sciences University of L'Aquila L'Aquila Italy; ^2^ European Center for Brain Research (CERC), Santa Lucia Foundation IRCCS Rome Italy; ^3^ Department of Otorhinolaryngology and Head & Neck Surgery, University Medical Center Utrecht Utrecht University Utrecht the Netherlands; ^4^ UMC Utrecht Brain Center Utrecht University Utrecht the Netherlands; ^5^ Department of Bioscience and Technology for Food, Agriculture and Environment University of Teramo Teramo Italy; ^6^ Leibniz Research Institute for Molecular Pharmacology (FMP), Campus Berlin‐Buch Berlin Germany; ^7^ Dompé Farmaceutici Spa L'Aquila Italy

**Keywords:** bioactive lipids, endocannabinoids, hearing loss, lipid signaling, organ of Corti

## Abstract

Cisplatin‐induced ototoxicity is a detrimental side effect of chemotherapy leading to hearing loss, for which no treatments are currently available. Despite the growing recognition of the endocannabinoid (eCB) system (ECS) as a significant contributor to different physiological and pathological processes, its role in hearing remains poorly investigated. To fill this knowledge gap, we performed a molecular profiling of the ECS in auditory hair cell (HC)‐like UB/OC1 cells derived from the mouse organ of Corti (OC), demonstrating the presence of the main eCBs‐binding receptors and metabolic enzymes along with the major eCBs (*N*‐arachidonoylethanolamine, AEA, and 2‐arachidonoylglycerol, 2‐AG) and additional eCB‐like compounds. Subsequently, we established an in vitro model of cisplatin‐induced ototoxicity, which was characterized by the downregulation of the HC marker myosin 7a (Myo 7a), and activation of nuclear factor kappa‐light‐chain‐enhancer of activated B‐cells (NF‐κB) associated with caspase‐3‐mediated cell death. In this model, we observed a downregulation of cannabinoid receptor 2 (CB_2_R), diacylglycerol lipase β (DAGLβ), and α/β hydrolase domain‐containing protein 6 (ABHD6), indicating a perturbation of the 2‐AG metabolic pathway. Furthermore, we validated the observed in vitro alterations in the OC of an in vivo model of cisplatin‐induced ototoxicity, thereby strengthening the physiological relevance of our findings. Finally, we demonstrated that pharmacological blockade of CB_2_R through SR144528 mitigates cisplatin‐induced HC damage via inhibition of caspase‐3 cleavage in UB/OC1 HC‐like cells, thereby providing novel mechanistic insights into the role of CB_2_R in ototoxicity. Overall, our study demonstrates the involvement of selective elements of the ECS in cisplatin‐induced ototoxicity, hence identifying novel potential biomolecular targets for chemotherapy‐related side effects.

## Graphical abstract

We performed a comprehensive molecular profiling of the endocannabinoid (eCB) system (ECS) in an auditory Hair cell (HC)‐like cell line (1). Afterwards, we developed suitable in vitro and in vivo models of cisplatin‐induced ototoxicity where we identified distinct components of the ECS (CB_2_R, DAGLβ and ABHD6) as molecular targets of the drug (2). Finally, pharmacological blockade of CB_2_R increased cell viability and decreased Caspase‐3 activation in vitro, thereby providing new insights into the molecular mechanisms of cisplatin‐induced auditory damage (3).

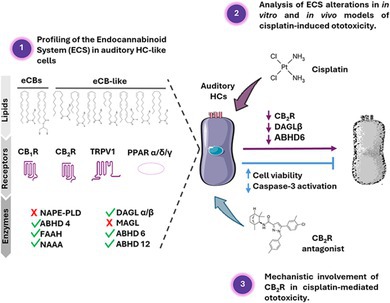

**Abbreviations**
2‐AG: 2‐Arachidonoylglycerol
AA: arachidonic acid
ABHD 4/6/12: α/β hydrolase domain‐containing protein
AEA: *N*‐arachidonoylethanolamine
ASC: apoptosis‐associated speck‐like protein
BSA: bovine serum albumin
CB_1_R: cannabinoid receptor 1
CB_2_R: cannabinoid receptor 2
CIS: cisplatin
COX: cyclooxygenase
DAG: diacylglycerol
DAGL α/β: diacylglycerol lipases α and β
DHEA: docosahexaenoylethanolamine
DMSO: dimethyl sulfoxide
eCBs: endocannabinoids
ECS: endocannabinoid system
ELISA: enzyme‐linked immunosorbent assay
EPEA: epoxyeicosatetraenoyl ethanolamide
EtNH_2_
: ethanolamine
FAAH: fatty acid amide hydrolase
FDA: Food and Drug administration
GAPDH: glyceraldehyde‐3‐phosphate dehydrogenase
GPCR: G‐protein coupled receptor
GSDMD: Gasdermin‐D
HC: hair cells
IC_50_
: half‐maximal inhibitory concentration
IHC: immunohistochemistry
IP_3_R: inositol‐3‐phosphate receptor
LEA: linoleoylethanolamide
LOX: lipoxygenase
LPS: lysophosphatidylserine
MAGL: monoacylglycerol lipase
MTT: 3‐(4,5‐dimethylthiazol‐2‐yl)‐2,5‐diphenyltetrazolium bromide
Myo7a: Myosin 7a
NAAA: *N*‐acylethanolamine‐hydrolyzing acid amidase
NAPE‐PLD: *N*‐acylethanolamines‐specific phospholipase D
NarPE: *N*‐arachidonoyl‐phosphatidylethanolamine
NF‐κB: Nuclear factor kappa‐light‐chain‐enhancer of activated B‐cells
NH: normal hearing
NLRP3: pyrin domain–containing‐3
OC: organ of Corti
OEA: *N*‐oleoylethanolamine
PBS: phosphate‐buffered saline
pe SPL: peak equivalent sound pressure level
PEA: *N*‐palmitoylethanolamine
PHARC: polyneuropathy, hearing loss, ataxia, retinosis pigmentosa, and cataract
POEA: palmitoleoyl ethanolamide
PPAR α, γ, δ: peroxisome proliferator‐activated nuclear receptors α, γ, δ
PUFA: polyunsaturated fatty acid
RT: room temperature
SEA: *N*‐stearoylethanolamine
SNHL: sensorineural hearing loss
TRPV1: transient receptor potential vanilloid receptor 1
VEH: vehicle

## 1 Introduction

Cisplatin is a platinum‐based compound that is widely used as a first line chemotherapeutic for treating several malignant tumors. Although it is quite efficient, it causes dose‐limiting adverse effects such as nephrotoxicity, neurotoxicity, and ototoxicity. Clinical ototoxicity is commonly presented as sensorineural hearing loss (SNHL), ear pain, tinnitus, and vestibular injury [1, 2]. To further worsen this unfavorable clinical picture, it has been shown that ototoxicity has a high incidence among cisplatin‐treated patients, with approximately 35% of adult cancer patients and 40%–60% of pediatric cancer patients developing hearing impairment [3]. To date, cisplatin‐induced hearing loss is known to be primarily caused by the injury to the cochlea, a fluid‐filled spiral structure located in the inner ear, responsible for sound perception [1]. Specifically, the cochlea is composed of a sensory epithelium named the organ of Corti (OC), which houses the hair cells (HCs). The latter are sensory receptors of sound, as they detect vibrations through the mechanical deflection of their stereocilia, which is then converted to neurochemical signals and transmitted to the rest of the auditory system up to the auditory cortex in the brain [4]. Unsurprisingly, HCs are thought to be the primary target of cisplatin‐induced hearing loss due to a multitude of factors including DNA damage, oxidative stress, and inflammatory processes [1, 5]. Although several studies have improved our understanding of cisplatin‐induced ototoxicity, the underlying molecular mechanisms have not yet been fully dissected. Thus, the development of targeted and effective therapies to prevent and/or counteract cisplatin‐induced ototoxicity remains largely limited.

The endocannabinoids (eCBs) are endogenous lipids that exert several biological functions under physiological and pathological conditions [6]. The primary eCBs include *N*‐arachidonoylethanolamine (or anandamide, AEA), and 2‐arachidonoylglycerol (2‐AG), both metabolized by their own enzymes that together form the endocannabinoid system (ECS) [6]. The biosynthesis of AEA occurs via the enzymes *N*‐acyl‐phosphatidylethanolamines‐specific phospholipase D (NAPE‐PLD) and α/β hydrolase domain‐containing protein 4 (ABHD4). Instead, AEA degradation is primarily catalyzed by fatty acid amide hydrolase (FAAH) and *N*‐acylethanolamine acid amidase (NAAA). 2‐AG is synthesized by diacylglycerol lipases α and β (DAGLα/β), and is hydrolyzed mainly by monoacylglycerol lipase (MAGL) and ABHD6/12 [6]. The eCBs exert their biological activity mainly through the binding to their target receptors, which include the G‐protein coupled receptors (GPCRs) known as cannabinoid receptor 1 (CB_1_R) and cannabinoid receptor 2 (CB_2_R), the transient receptor potential vanilloid type‐1 (TRPV1), and the peroxisome proliferator‐activated receptors α, δ, and γ (PPARα/δ/γ) [7]. In addition to classical eCBs, other eCB‐like *N*‐acylethanolamines (NAEs) have been shown to impact AEA and 2‐AG signaling [6]. Among them, *N*‐palmitoylethanolamine (PEA), *N*‐oleoylethanolamine (OEA), *N*‐linoleoylethanolamine (LEA), *N*‐stearoylethanolamine (SEA), *N*‐palmitoleoylethanolamine (POEA), *N*‐epoxyeicosatetraenoylethanolamine (EPEA), and *N*‐docosahexaenoylethanolamine (DHEA) have attracted attention. However, knowledge on metabolism and signaling of these eCB‐like compounds remains largely limited compared to eCBs. To date, only a few studies have interrogated the role of the ECS in cisplatin‐induced ototoxicity [8] and they are mostly limited to some eCBs‐binding receptors such as CB_1_R [9], CB_2_R [8, 10] and TRPV1 [11]. Of note, the available data remains fragmentary and inconclusive. For instance, only one study has investigated the impact of cisplatin on cochlear CB_2_R, and it was limited to gene expression [12]. Moreover, mechanistic studies on the same receptor so far rely only on the effect of JWH015 [10, 13], which is not the most selective CB_2_R agonist [14] Therefore, all available data warrant a cautious interpretation.

On this basis, in the present investigation, we sought to fill the knowledge gap on cochlear ECS by performing a comprehensive analysis of the ECS in auditory HC‐like cells derived from the mouse OC. Furthermore, we developed in vitro and in vivo models of cisplatin‐induced ototoxicity suitable to explore the molecular underpinnings of HC damage, thereby identifying distinct ECS components in HCs as targets of cisplatin.

## 2 Materials and Methods

### 2.1 Cell Culture

Immortalized auditory HC‐like UB/OC1 cells derived from the mouse organ of Corti were purchased from Cancertools.org (Cat. no. 153622), and were cultured in minimal essential medium (MEM) with Earl's salts supplemented with 10% fetal calf serum (Cytiva), 1% glutamine (Sial, cat. no. SIAL‐L‐Glu), 50 U/mL γ‐interferon (Pepro Tech, cat. no. 315‐05). Fresh γ‐interferon was replaced every 65 h. Cell culture was performed at 33°C in an incubator with 5% CO_2_. Trypsin 1X (Euroclone, cat. no. ECB3052D) was used for passaging cells.

### 2.2 Cisplatin‐Induced Ototoxicity

Cells were seeded at a density of 25 000 cells/cm^2^ in a 96‐well plate for 24 h, and then treated with increasing concentrations of cisplatin (Sigma Aldrich, cat. no. P4394): 2.5, 6.25, 12.5, 25, and 50 μM [15, 16]. A stock of 0.2 mg/mL of cisplatin solution was first prepared in physiological solution (0.9% sodium chloride) and then diluted to the desired concentrations in complete medium. Control cells were treated with vehicle (veh) alone. Cell treatment lasted 24 h, as previously reported [17, 18].

### 2.3 MTT Cell Viability Assay

Cells were seeded at a density of 25 000 cells/cm^2^ on a 96‐well plate and cultured for 24 h. Afterward, cisplatin or vehicle treatment was performed and then 5 mg/mL 3‐(4,5‐dimethylthiazol‐2‐yl)‐2,5‐diphenyltetrazolium bromide (MTT) (Merck, cat. no. M2128‐1G) in 0.1 M phosphate‐buffered saline (PBS1X) was added to the cells, which were incubated for 2 h at 33°C. Acidified isopropanol (2‐propanol‐0.04 N HCl) was used in a 1:1 ratio to dissolve the formazan crystals, and the plate was immediately read with TECAN Magellan Pro v7.4 microplate reader (Promega; cat. no. 30172786) at 570 nm and 630 nm (reference absorbance). Values of cell viability were then calculated by subtracting the 630 nm OD from the 570 nm OD and were expressed as folds over control. Half‐maximal inhibitory concentration (IC_50_) of cisplatin at 30 μM was calculated by linear regression analysis of MTT data and was used for subsequent analyses.

### 2.4 Trypan Blue Dye Exclusion Test

Trypan blue (Sigma‐Aldrich, cat no. T8154) dye exclusion test was performed as an additional method to assess cell viability. Briefly, cells were trypsinized and collected, then the cell suspension was mixed with 0.1% trypan blue at a ratio of 1:2.5. Cell counting was performed manually using a hemocytometer. Cells that were not stained with trypan blue were counted as live cells, and cells stained with trypan blue were counted as dead cells. Cell viability was then expressed as folds over vehicle.

### 2.5 Protein Extraction and Quantification

Cells were cultured and treated with 30 μM cisplatin for 24 h in a 75 cm^2^ culture flasks. After treatments, cells were trypsinized and pelleted. The collected cell pellets were resuspended in a lysis buffer (50 mM Tris–HCl pH 7.5, 1% Triton X‐100, 0.1% SDS, EDTA 5 Mm, Halt Protease and Phosphatase Inhibitor Cocktail, Thermo Fisher Scientific, Waltham, MA, USA and QS dH2O). The lysates were kept on ice for 30 min and then centrifuged at 13 200 rpm for 20 min at 4°C. The supernatant was collected and then stored at −80°C. Bradford (BioRad, cat. no. 5000006) assay was then used to quantify the proteins. Mouse Brain, liver, and heart were used as positive controls for appropriate proteins and their extraction followed the same procedure.

### 2.6 Western Blotting

60 μg of protein extracts were loaded in Bolt 4%–12% Bis‐Tris Plus (Thermofisher Scientific, cat. no. NW04120BOX) and transferred using iBlot 3 Transfer Stacks, midi, PVDF (Thermo Fisher Scientific, cat. no. IB34001) in iBlot 3 dry transfer system. Immunoblots were performed by diluting primary antibodies in 5% non‐fat dry milk (PanReac AppliChem ITW Reagents, cat. no. A0830) or 5% Bovine serum albumin (BSA) (Merck, cat. no. A3059) for detecting the following components: Myo7a, CB_1/2_R, TRPV1, PPARα/γ/δ, NAPE‐PLD, NAAA, FAAH, ABHD 4, DAGLα/β, ABHD6/12, nucleotide‐binding domain, leucine‐rich–containing family, pyrin domain–containing‐3 (NLRP3), apoptosis‐associated speck‐like protein (ASC), gasdermin‐D (GSDMD), N‐GSDMD, pro‐caspase‐1, cleaved‐Caspase‐1, NF‐κB, α/β tubulin, lamin A/C, pro‐Caspase‐3, cleaved‐Caspase‐3, glyceraldehyde‐3‐phosphate dehydrogenase (GAPDH). The detailed list of primary antibodies used is shown in Table 1. Afterwards, membranes were incubated with specific HRP‐conjugated secondary antibody (anti‐rabbit/anti‐mouse/anti‐goat), diluted in 5% nonfat dry milk in TBST. The detailed list of secondary antibodies used is shown in Table 2. The membranes were then developed using Super Signal West Pico Plus (Thermo Fisher Scientific, cat. no. 34580) or Super Signal West Atto Ultimate Sensitivity (Thermo Fisher Scientific, cat. no. 38555) chemiluminescent substrates and the bands were detected using a ChemiDoc XRS plus imaging system (Bio‐Rad Laboratories; RRID:SCR_019690). The densitometry analysis was performed using Image J software (RRID:SCR_003070).

**TABLE 1:** List of primary antibodies used in western blotting.

Primary antibody	Supplier and catalog number	Host species	Dilution	Dilution medium	Chemiluminescent substrate used
CB_1_R	Abcam‐ab259323	Rabbit	1:1000	Nonfat dry milk 5%	Super Signal West Pico Plus
CB_2_R	Cayman Chemical 101550	Rabbit	1:200	Nonfat dry milk 5%	Super Signal West Pico Plus
TRPV1	OriGene TA336871	Rabbit	1:1000	Nonfat dry milk 5%	Super Signal West Pico Plus
PPARα	Sigma Aldrich SAB4502260	Rabbit	1:500	Nonfat dry milk 5%	Super Signal West Pico Plus
PPARδ	Invitrogen PA1‐823A	Rabbit	1:500	Nonfat dry milk 5%	Super Signal West Atto Ultimate Sensitivity
PPARγ	Cell Signaling 2443	Rabbit	1:500	BSA 5%	Super Signal West Atto Ultimate Sensitivity
NAPE‐PLD	Cayman Chemical 10305	Rabbit	1:200	Nonfat dry milk 5%	Super Signal West Atto Ultimate Sensitivity
ABHD4	Invitrogen PA5‐75867	Rabbit	1:500	Nonfat dry milk 5%	Super Signal West Pico Plus
FAAH	Abcam Ab‐54615	Mouse	1:500	Nonfat dry milk 5%	Super Signal West Atto Ultimate Sensitivity
NAAA	Invitrogen PA5‐48061	Goat	1:500	Nonfat dry milk 5%	Super Signal West Atto Ultimate Sensitivity
DAGLα	Santa Cruz SC‐390409	Mouse	1:100	Nonfat dry milk 5%	Super Signal West Atto Ultimate Sensitivity
DAGLβ	Cell Signaling 12574 (D4P7C)	Rabbit	1:1000	BSA 5%	Super Signal West Atto Ultimate Sensitivity
MAGL	Abcam Ab24701	Rabbit	1:200	Nonfat dry milk 5%	Super Signal West Atto Ultimate Sensitivity
ABHD6	Invitrogen PA5‐101396	Rabbit	1:500	Nonfat dry milk 5%	Super Signal West Atto Ultimate Sensitivity
ABHD12	Antibodies.com ABIN3183122	Rabbit	1:500	Nonfat dry milk 5%	Super Signal West Atto Ultimate Sensitivity
GAPDH	Invitrogen MA1‐16757	Mouse	1:1500	Nonfat dry milk 5%	Super Signal West Pico Plus
Myosin VII A (Myo7a)	Proteus Biosciences‐PTS‐25‐6790‐C050	Rabbit	1:200	Nonfat dry milk 5%	Super Signal West Atto Ultimate Sensitivity
NF‐κB	Cell Signaling 8242	Rabbit	1:1000	BSA 5%	Super Signal West Pico Plus
α/β Tubulin	Cell Signaling 2148S	Rabbit	1:1000	Nonfat dry milk 5%	Super Signal West Pico Plus
Lamin A/C	Cell Signaling 4777S	Mouse	1:1000	BSA 5%	Super Signal West Pico Plus
NLRP3	Cell Signaling 15101	Rabbit	1:500	BSA 5%	Super Signal West Atto Ultimate Sensitivity
ASC	Cell Signaling 67824 (D2W8U)	Rabbit	1:1000	BSA 5%	Super Signal West Atto Ultimate Sensitivity
GSDMD	Cell Signaling 39754 (E9S1X)	Rabbit	1:1000	BSA 5%	Super Signal West Atto Ultimate Sensitivity
Caspase‐1	Cell Signaling 83383	Rabbit	1:500	BSA 5%	Super Signal West Atto Ultimate Sensitivity
Caspase‐3	Cell signaling 9662S	Rabbit	1:1000	Nonfat dry milk 5%	Super Signal West Atto Ultimate Sensitivity

**TABLE 2:** List of secondary antibodies used in western blotting.

Secondary antibody	Supplier and catalog number	Host species	Dilution	Dilution medium
Anti‐Rabbit IgG (H + L) HRP	Invitrogen 31460	Goat	1:10 000	Nonfat dry milk 5%
Anti‐Mouse IgG (H + L) HRP	Invitrogen 31430	Goat	1:10 000	Nonfat dry milk 5%
Anti‐goat HRP	Antibodies.com AI7358	Rabbit	1:5000	Nonfat dry milk 5%

### 2.7 Nuclear/Cytoplasmic Fractionation

To investigate NF‐κB activation, we measured its sub‐cellular protein levels [19]. To this end, fresh cell pellets were extracted, and the respective nuclear and cytoplasmic protein fractions were separated. The separation was performed using the NE‐PER kit (Thermofisher, cat. no. 78833) according to the manufacturer's instructions. 30 μg of cytoplasmic and nuclear extracts were loaded as previously described. As housekeeping controls, α/β tubulin was used for the cytoplasmic fractions, whereas lamin A/C was used for the nuclear fractions.

### 2.8 IL‐1β ELISA Assay

After treatment of UB/OC1 cells with cisplatin or vehicle, released IL‐1β levels were measured using enzyme‐linked immunosorbent assay (ELISA) with the mouse IL‐1β ELISA kit (Invitrogen, cat. no. 88‐7013‐22). The absorbance was measured at 450 nm and 570 nm (reference absorbance) using TECAN Magellan Pro v7.4 microplate reader (Promega, cat. no. 30172786). The absorbance at 450 nm was subtracted from that at 570 nm, and the resulting absorbance value was calculated from standard curves plotted through the curve fitting software CurveExpert 1.4 (Hyams Development).

### 2.9 Ultra‐Performance Liquid Chromatography–Tandem Mass Spectrometry (UHPLC–MS/MS)

The analysis of the eCBs profile was performed as we reported earlier [20] with minor modifications. Briefly, samples were homogenized and extracted by adding 125 μL of MeOH and 200 mM formic acid to a Precellys 24 tissue homogenizer (Bertin, Montigny‐le‐Bretonneux, France). Internal standards (AEA‐d4, PEA‐d4, 2‐AG‐d8) were spiked into the extraction solvent at a final concentration of 5 ng/mL in order to normalize the data. Subsequently, sample clean‐up was performed using OMIX C18 tips from Agilent Technologies (Santa Clara, CA, USA). UHPLC–MS/MS analysis was performed by a Waters UPLC Acquity H‐Class (Waters, Milford, MA, USA), coupled with a Qtrap 4500 from Sciex (Toronto, ON, Canada), equipped with a Turbo V source operating in electrospray ionization source and operating in positive mode (ESI+). Separation of the target analytes was performed by a Waters Acquity BEH C18 column (100 × 2.1 mm, packed with 1.8 μm particles), using H_2_O and acetonitrile: methanol (MeOH:CAN) 50:50 (v:v)—both with 0.01% formic acid—as mobile phases A and B, respectively [20]. Lipid levels were then calculated as pmol/10^6^ cells.

### 2.10 Immunocytochemistry

Cells were seeded on coverslips coated with 1.5% gelatin (Sigma Aldrich, cat. no. G‐9391) for 24 h and cisplatin treatments were performed as described above. Cells were then fixed with Paraformaldehyde (PFA) 4% (Sigma‐Aldrich, cat. no. 100496) and then blocked for unspecific bindings using BSA 5% for 1 h at room temperature (RT). Afterward, cells were incubated overnight with primary antibodies at 4°C to detect the following proteins: Myo7a, CB_2_R, DAGLβ, ABHD6. The detailed list of primary antibodies used for immunofluorescence is reported in Table 3. Cells were then incubated with secondary antibodies (anti‐rabbit IgG conjugated to red fluorescent dyes) (Alexa Fluor goat‐anti Rabbit 594, Invitrogen, cat. no. A11012), diluted 1:300 in PBS 1X at 37°C for 2 h. Bisbenzimide nuclear dye (Hoechst) was used to label the nuclei. The coverslips were then mounted using gelatin and visualized using a TCS SP5 confocal microscope (Leica). The parameters of gain and offset were the same for both control and treated samples. The fluorescence intensity profile was plotted as a histogram by manually selecting regions of interest (ROI), which calculated pixel intensity values on the basis of red fluorescence channel data. The parameters of ROIs were set the same for both control and treated conditions.

**TABLE 3:** List of primary antibodies used in immunocytochemistry.

Primary antibody	Supplier and catalog number	Dilution
Myosin VII A (Myo7a)	Proteus Biosciences‐ PTS‐25‐6790‐C050	1:200
CB_2_R	Cayman Chemical 101550	1:50
DAGLβ	Invitrogen PA5‐26331	1:50
ABHD6	Invitrogen PA5‐101396	1:100

### 2.11 SiR‐8 Live Cell Confocal Imaging

CB_2_R localization was assessed by live confocal imaging using the highly potent, fluorescent CB_2_R agonist SiR‐8 [21]. Briefly, cells were seeded in 8‐well IBIDI chambers (Ibidi, cat. no. 80826) at a density of 10 000 cells/well for 24 h, and treated with cisplatin as described above. Then, cells were incubated with 0.8 μM SiR‐8, and time‐lapse confocal imaging was performed using a TCS SP5 confocal microscope (Leica). Specifically, live imaging was performed by recording one image every 15 s for 20 min setting the same gain and offset parameters between the experimental groups. Images were then exported as TIFF files, and a video was created using the Clipchamp software (Microsoft, Redmond, WA, USA) to visualize time‐related SiR‐8 signal. The fluorescence intensity profile was plotted as a histogram by manually selecting regions of interest (ROIs), which calculate pixel intensity values on the basis of red channel data. The parameters of ROIs were the same for both control and treated conditions [22].

### 2.12 Treatment on UB/OC1 Cells With SR144528

Cells were seeded at a density of 25 000 cells/cm^2^ for 24 h and then treated with selected concentrations of SR144528 [23, 24, 25] (Sigma Aldrich cat. no. SML1899) (2 μM) for 1 h in culture medium without FCS. A stock solution of 8 mM of SR144528 was first prepared in 100% Dimethyl sulfoxide (DMSO) and was then diluted to the desired concentration in the culture medium without FCS. The final DMSO concentrations did not exceed 0.03%. Afterward, cells were treated with the IC_50_ concentration of cisplatin (30 μM) for 24 h as previously reported.

### 2.13 Animals

Seven adult male and female C57BL/6J mice (Charles River Laboratories) (RRID:SCR_003792), aged 8–12 weeks, were used for the study. Animals were housed in individually ventilated cages (IVC) with food and water under standard laboratory conditions. All animal experimental procedures were approved by the Dutch Central Authority for Scientific Procedures on Animals (CCD: AVD11500202418278). All investigations were conducted in compliance with the ARRIVE guidelines, including the 3R (Replacement, reduction, and refinement) principle.

### 2.14 Animal Model of Cisplatin‐Induced Ototoxicity

The mouse model of cisplatin‐induced ototoxicity was adapted from [26]. Briefly, animals were administered a single dose of 200 mg/kg furosemide intraperitoneally (i.p.) followed by 2 mg/kg cisplatin i.p. Hydration support was provided by administering lactated ringers' solution (Braun) 3 days after cisplatin treatment if weights dropped below 5% from their day 0 weight. This supportive care was given to maintain electrolyte balance and support renal functions. Animals were weighed throughout the experiment to monitor their health. Hearing loss was assessed using click‐evoked auditory brainstem responses (ABRs) at the end of the experiment. The schematic representation of the experimental design is provided in Figure S1.

### 2.15 Auditory Brainstem Responses

Auditory brainstem responses (ABRs) were performed as described previously [27, 28] to confirm normal hearing (NH) before the start of the experiments and at the endpoint of the ototoxicity protocols. Briefly, animals were maintained under anesthesia using isoflurane in a soundproof and electrically shielded box (52 × 34 × 28 cm). The animals were placed on a heating pad throughout the procedure. Subdermal needle electrodes (27ga needle, 13 mm) were placed behind the right pinna (active), on the skull (reference), and hind limb (ground). A TDT3 system (Multi‐I/O processor RZ6; Tucker‐Davis Technologies, Alachua, FL, USA) was used to generate acoustic stimuli of 20 μs‐monophasic clicks. The acoustic stimuli were presented using a speaker (Bowers and Wilkins, CCM683; 8 Ω; 25–130 W) kept at 5 cm from the left ear. The signals were pre‐amplified using a 5113‐pre‐amplifier (Princeton Applied Research, Oak Ridge, TN, USA) with an amplification of × 5000 and a band pass filter of 0.1–10 kHz. The digitized signals from the TDT3 system were then used for analyses (100 kHz sampling rate, 24‐bit sigma‐delta converter). The responses were then analyzed using MATLAB software. Stimulus intensity was applied in 10 dB attenuation steps starting with the maximum sound level at approximately 105 dB peak equivalent sound pressure level (peSPL), and ABRs were recorded until there were no responses. The threshold criteria were determined by the interpolated sound level, where the amplitude of the largest wave measured was 0.3 μV. A threshold shift of > 10 dB was employed as the criterion for indicating the onset of functional hearing impairment, as previously described [17, 29].

### 2.16 Tissue Processing and Histology

For histological analysis of cochlear tissues, the otic capsule containing the cochlea was collected immediately after euthanizing the animal and fixed with paraformaldehyde (PFA) 4% (Sigma‐Aldrich, cat. no. 100496) for 48 h at 4°C. Once the fixation was complete, the samples were transferred to a decalcification solution containing 0.12 M EDTA (Invitrogen, cat. no. 15575020) in PBS 1X and maintained in slow agitation for 7–9 days at 4°C. Cryopreservation of the specimens was performed by stepwise sucrose (Sigma‐Aldrich, cat. no. S7903) gradients of 5%, 15%, and 30% for 1 day each. Afterwards, they were processed for mid‐modiolar cryosectioning by embedding the decalcified tissue in Tissue Tek OCT (VWR, cat. no. 361603E), and were stored at −20°C until sectioning. Mid‐modiolar sections of 10 μm were obtained using a Leica CM1850 cryostat (Nussloch, GmbH, Germany) and mounted on coated Superfrost‐PLUS slides for immunostaining.

### 2.17 Immunohistochemistry

Immunohistochemistry (IHC) was performed to investigate the alterations of CB_2_R, DAGLβ, and ABHD6 in the OC of cisplatin‐treated and control animals. To this end, mid‐modiolar sections were first allowed to thaw at RT for 20 min. Then, the sections were washed with PBS 1X for 5 min. Afterward, unspecific bindings were blocked with suitable blocking buffers for each protein (see Table 4). After the blocking step, slides were incubated overnight with primary antibodies at 4°C (detailed in Table 4). The following day, slides were washed and incubated with secondary antibodies (Alexafluor 488, Molecular Probes, Invitrogen, Carlsbad, CA, USA) for 90 min at RT (detailed in Table 4). Slides were then imaged using a Stellaris 8 STED confocal microscope. A z‐stack containing ~4–6 planes with a step size of 0.5 μm was set. The parameters of gain and offset were the same for both control and treated samples. Fluorescence intensity in each OC was analyzed using Image J software and is expressed as folds over control.

**TABLE 4:** List of primary and secondary antibodies used for immunohistochemistry.

Protein	Blocking solution	Primary antibodies and dilutions	Secondary antibodies and dilutions
CB_2_R	BSA 5% + PBS‐(Triton 0.1%)	Anti‐CB_2_R, Cayman chemicals; #101550, 1:50	Goat‐ anti rabbit Alexafluor 488, 1:300
DAGLβ	Goat serum 10% + PBS‐Triton (0.1%)	Anti‐DAGLβ, Invitrogen; #PA5‐26331, 1:100	Goat‐ anti rabbit Alexafluor 488, 1:500
ABHD6	Goat serum 10% + PBS‐Triton (0.1%)	Anti‐ABHD6, Invitrogen; # PA5‐101396, 1:100	Goat‐ anti rabbit Alexafluor 488, 1:500

### 2.18 Statistical Analysis

Statistical analysis was performed using two‐tailed unpaired Student's *t*‐test, one‐way ANOVA, or two‐way ANOVA tests followed by Bonferroni *post hoc* comparison. The detailed statistical test and the number of biological replicates (*n*) for each experiment are included in the corresponding figure legends. *p* < 0.05 was considered statistically significant. Grubb's test was used to determine any outliers. All data were tested for normality using the Shapiro–Wilk test. GraphPad Prism 8 software (GraphPad Software Inc., San Diego, CA, USA) was used to perform statistical analysis.

## 3 Results

### 3.1 Identification of the Main ECS Components in UB/OC1 Cells

As a first step, a systematic profiling of the ECS was performed in UB/OC1 cells. The primary receptor targets and metabolic enzymes of eCBs were assessed at a protein level by using robust positive controls, shown in Tables S1 and S2 with the relevant references.

**FIGURE 1:**
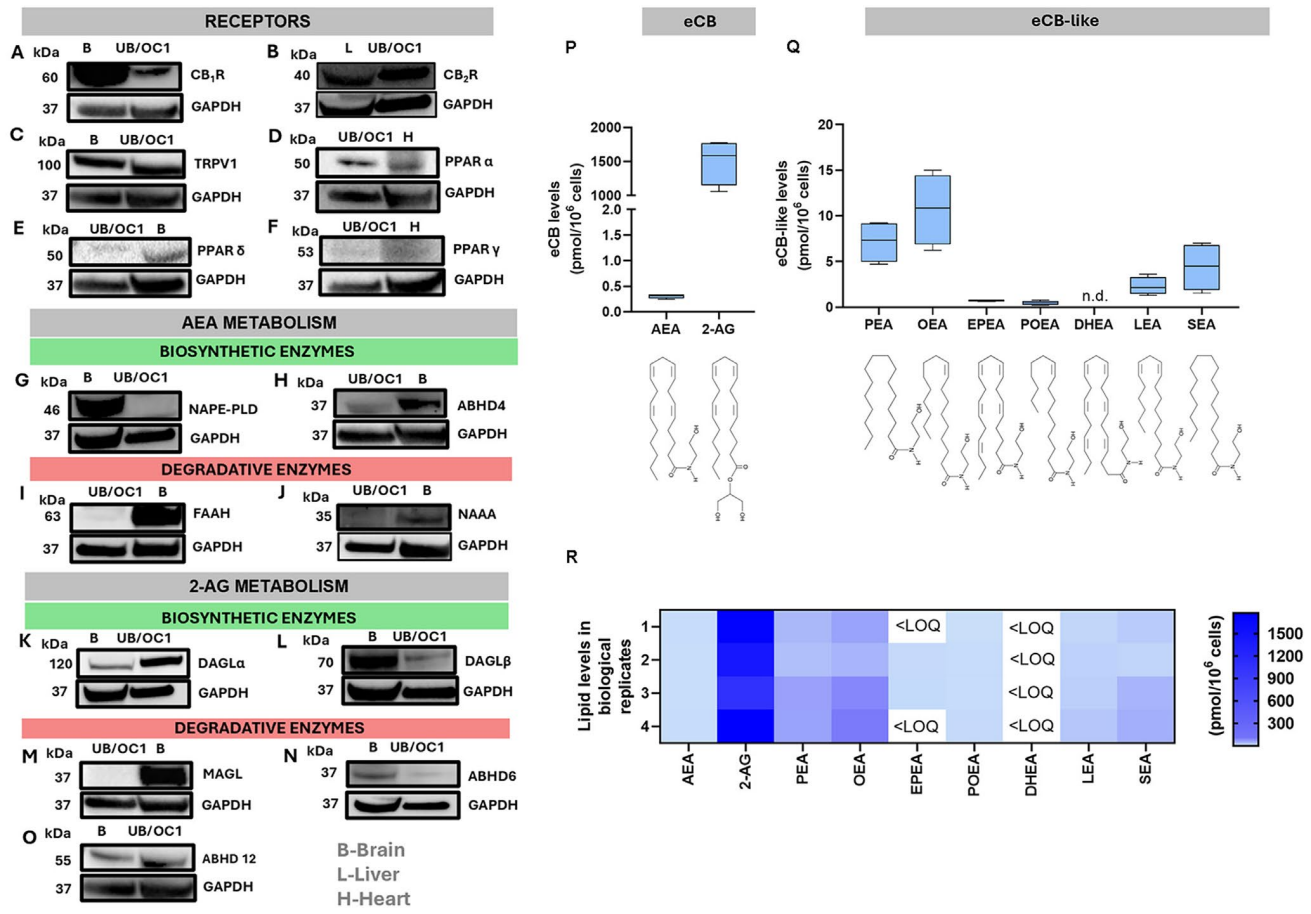
Identification of the principal ECS elements in UB/OC1 cells. Western blots of eCB receptors (A) CB_1_R, (B) CB_2_R, (C) TRPV1, (D) PPARα, (E) PPARδ, and (F) PPARγ. Western blots of the AEA biosynthetic enzymes (G) NAPE‐PLD and (H) ABHD4, and of the AEA degradative enzymes (I) FAAH and (J) NAAA. Western blots of the primary 2‐AG metabolic enzymes (K) DAGLα, (L) DAGLβ, and (M) MAGL, and of the additional 2‐AG hydrolases (N) ABHD6 and (O) ABHD12. Each protein is shown alongside positive controls from mouse tissues with respective GAPDH housekeeping. The whole Western blot bands are reported in Figures S2–S16. (P) Endogenous levels of AEA and 2‐AG and of (Q) eCB‐like compounds in UB/OC1 cells quantified by UHPLC–MS/MS. The data are presented as a box plot with whiskers ranging from min to max values. Each eCB is presented alongside its chemical structure. (R) Levels of eCBs and eCB‐like compounds in UB/OC1 cells are represented as a heatmap (*n* = 4). 2‐AG, 2‐arachidonoylglycerol; AEA, *N*‐arachidonoylethanolamine; ABHD4/6/12, α/β hydrolase domain‐containing protein; B, brain; CB_1_R, cannabinoid receptor 1; CB_2_R, cannabinoid receptor 2; DAGLα/β, diacylglycerol lipases α and β; DHEA, docosahexaenoylethanolamine; EPEA, *N*‐epoxyeicosatetraenoylethanolamine; FAAH, fatty acid amide hydrolase; GAPDH, glyceraldehyde‐3‐phosphate dehydrogenase; H, heart; L, liver; LEA, *N*‐linoleoylethanolamine; MAGL, monoacylglycerol lipase; NAPE‐PLD, *N*‐acyl‐phosphatidylethanolamines‐specific phospholipase D; NAAA, *N*‐acylethanolamine acid amidase; OEA, *N*‐oleoylethanolamine; PEA, *N*‐palmitoylethanolamine; POEA, *N*‐palmitoleoylethanolamine; PPARα/γ/δ, peroxisome proliferator‐activated nuclear receptors α, γ, δ; SEA, *N*‐stearoylethanolamine; TRPV1, transient receptor potential vanilloid receptor 1.

Firstly, we demonstrated that UB/OC1 cells express the main eCBs‐binding receptors, namely CB_1_R (Figure 1A), CB_2_R (Figure 1B), and TRPV1 (Figure 1C), along with the nuclear receptors PPARα/δ/γ (Figure 1D–F). Then, we analyzed the metabolic enzymes of the two major eCBs AEA and 2‐AG, because the metabolism and signaling of other eCB‐like lipids remain poorly understood [6, 30, 31, 32, 33]. The major biosynthetic enzyme of AEA, NAPE‐PLD, was not detected in UB/OC1 cells (Figure 1G). Instead, the biosynthetic enzyme ABHD4 (Figure 1H) and the degradative enzymes FAAH (Figure 1I) and NAAA (Figure 1J) were found to be expressed. As for 2‐AG, we found that UB/OC1 cells express the key enzymes of 2‐AG biosynthesis, DAGLα and DAGLβ (Figure 1K,L), whereas the major 2‐AG hydrolase MAGL was not detected (Figure 1M). Interestingly, ABHD6 (Figure 1N) and ABHD12 (Figure 1O), which are known as relevant players in 2‐AG degradation, were detected in UB/OC1 cells.

We then measured the endogenous content of eCBs and congeners (PEA, OEA, LEA, SEA, POEA, EPEA and DHEA) through targeted lipidomics. AEA and 2‐AG were found to be present in UB/OC1 cells, with 2‐AG levels (~1503.027 pmol/10^6^ cells) being much higher than those of AEA (~0.302 pmol/10^6^ cells), as shown in Figure 1P. We were also able to detect eCB‐like compounds PEA (~7.160 pmol/10^6^ cells), LEA (~2.301 pmol/10^6^ cells), OEA (~10.722 pmol/10^6^ cells), SEA (~4.403 pmol/10^6^ cells), POEA (~0.477 pmol/10^6^ cells), and EPEA (~0.353 pmol/10^6^ cells), but not DHEA. These findings are shown in Figure 1Q, and in Figure 1R as a heatmap.

### 3.2 Development of the In Vitro Model of Cisplatin‐Induced Ototoxicity

**FIGURE 2:**
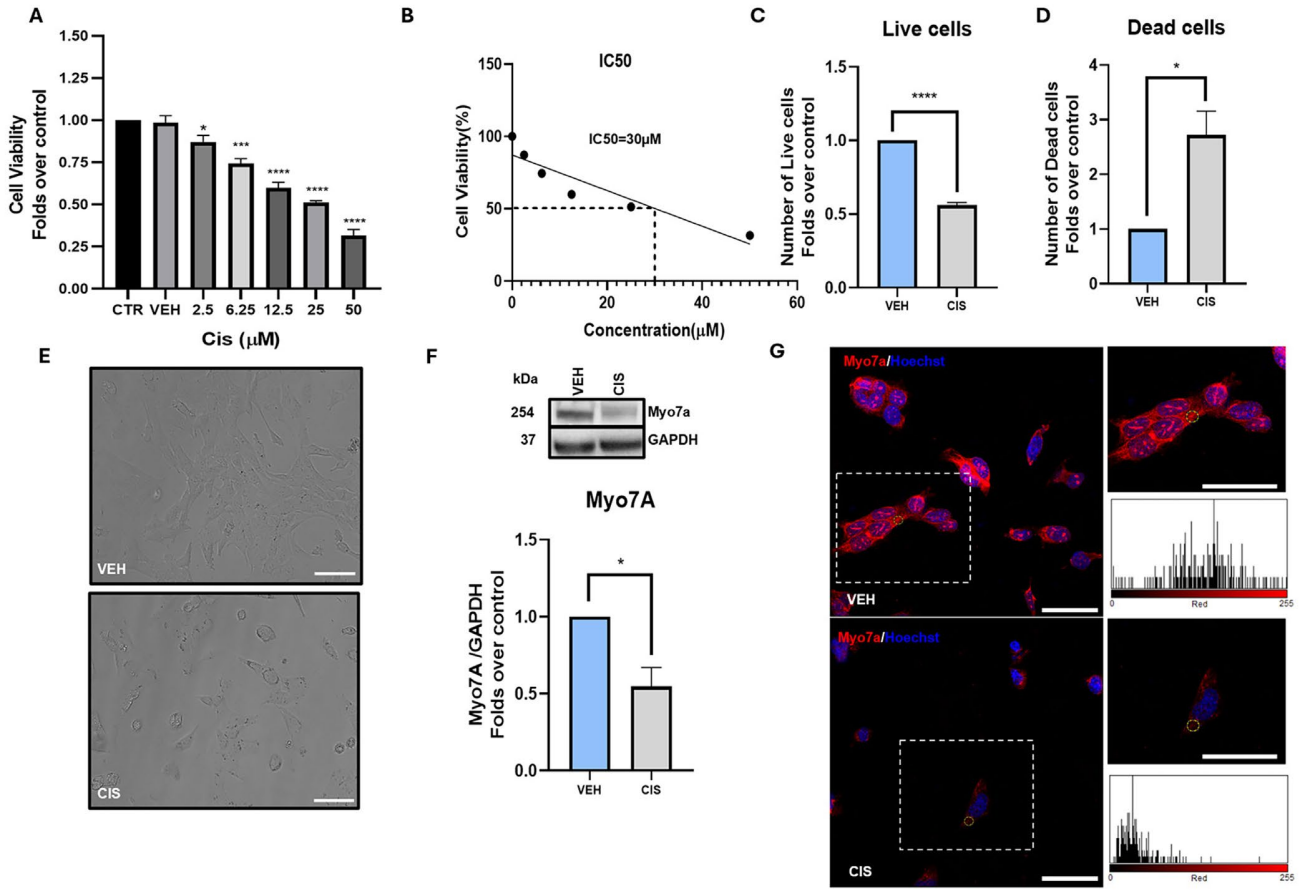
Cell viability and Myo7a quantitation in cisplatin‐treated UB/OC1 cells. (A) Cell viability of UB/OC1 cells upon treatment with increasing concentrations of cisplatin, determined by MTT assay. Statistical analysis was performed by one‐way ANOVA test with Bonferroni *post hoc* comparison (*n* = 3) (*p ≤ 0.05, ***p ≤ 0.001 and ****p ≤ 0.0001 vs control). (B) IC50 determination of cisplatin from MTT assay using linear regression. (C, D) Counting of live and dead cells respectively by using trypan blue exclusion test in VEH and CIS treated cells. Statistical analysis was performed using two‐tailed unpaired Student's *t*‐test (*n* = 3) (*p ≤ 0.05, ****p ≤ 0.0001 vs VEH). (E) Representative phase‐contrast images of VEH and CIS treated UB/OC1 cells, scale bar: 100 μm. (F) Western blot bands of Myo7a with respective GAPDH housekeeping in VEH and CIS treated cells and their densitometric quantification. Statistical analysis was performed using student's *t*‐test (*n* = 3) (*p ≤ 0.05 vs VEH). Whole western blot bands are reported in Figure S17. (G) Representative confocal microscopy images of Myo7a (red) counterstained with Hoechst nuclear dye (blue), scale bar: 50 μm. Although dotted boxes show zoomed‐in views of UB/OC1 cells, and the corresponding histograms are representative of the yellow circle‐marked areas. Data are presented as mean ± SEM. CIS, cisplatin; CTR, control; GAPDH, glyceraldehyde‐3‐phosphate dehydrogenase; IC_50_, half‐maximal inhibitory concentration; MTT, 3‐(4,5‐dimethylthiazol‐2‐yl)‐2,5‐diphenyltetrazolium bromide; Myo7a, myosin 7a; VEH, vehicle.

After a systematic characterization of the main ECS components in UB/OC1 cells, we aimed to study the possible implications of cisplatin for eCB signaling. To this end, we developed an in vitro model of cisplatin‐induced ototoxicity. Briefly, the cells were treated with a range (2.5 μM to 50 μM) of cisplatin concentrations for 24 h, and cell viability was assessed via the MTT assay (Figure 2A). A significant dose‐dependent decrease in viability of UB/OC1 cells was observed upon cisplatin treatment, compared to control and vehicle‐treated cells (Figure 2A). Moreover, no difference was observed between control and vehicle‐treated cells; therefore, the latter cells were used as control for all further experiments. On the basis of MTT data, we determined the half‐maximal inhibitory concentration (IC_50_) of cisplatin as 30 μM, which was selected as the suitable dose for subsequent experiments (Figure 2B). The cytotoxicity of 30 μM cisplatin was further confirmed by the trypan blue dye exclusion test, which indeed showed both a significant decrease in the number of live cells (0.5‐fold over vehicle; *p* < 0.0001) (Figure 2C) and a significant increase in the number of dead cells (3‐fold over vehicle; *p* = 0.0166) (Figure 2D) upon cisplatin treatment. Phase‐contrast images further supported the cytotoxicity of cisplatin (Figure 2E).

To further support the validity of our model for cisplatin‐induced ototoxicity, myosin 7a (Myo7a) levels were assessed in UB/OC1 cells in response to cisplatin‐induced damage. Indeed, Myo7a is a distinct marker for sensory HCs in the organ of Corti, and its downregulation has been demonstrated in animal models of cisplatin‐induced ototoxicity [34, 35]. Firstly, we confirmed the expression of Myo7a in UB/OC1 cells through Western blotting (Figure 2F) and immunofluorescence (Figure 2G), as previously reported [36]. Notably, we observed a significant reduction in the protein levels of Myo7a after cisplatin treatment compared to vehicle (*p* = 0.0205) (Figure 2F), a finding supported by immunofluorescence staining (Figure 2G).

### 3.3 Activation of NF‐ κB and Caspase‐3 Upon Cisplatin‐Induced Ototoxicity

**FIGURE 3:**
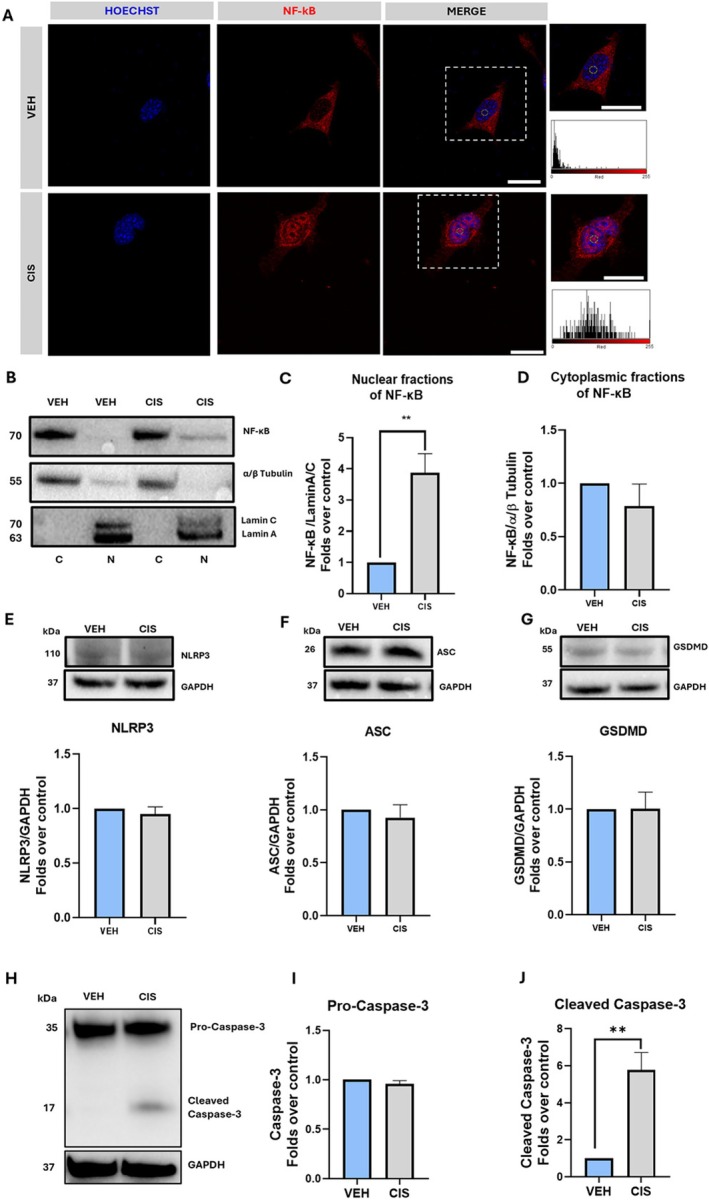
NF‐κB and downstream signaling pathways in cisplatin‐treated UB/OC1 cells. (A) Representative confocal microscopy images of NF‐κB (red) counterstained with Hoechst nuclear dye (blue) in VEH and CIS‐treated cells; scale bar: 25 μm. White dotted boxes show zoomed‐in views of UB/OC1 cells, and the corresponding fluorescence intensity histograms are representative of the yellow circle‐marked areas. (B) Representative western blot images of NF‐κB in cytoplasmic (C) and nuclear fractions (D) with respective α/β tubulin as a cytoplasmic housekeeping and Lamin A/C as a nuclear housekeeping. (C, D) Densitometric quantification of the western blot bands of NF‐κB in nuclear fraction (C) and cytoplasmic fraction (D). (E–G) Representative western blot bands of NLRP3, ASC, and GSDMD with respective GAPDH housekeeping and densitometric quantification of the western blot bands of NLRP3 (E), ASC (F), and GSDMD (G) normalized over GAPDH. (H) Representative Western blot bands of proteins pro‐caspase‐3 and cleaved‐caspase‐3 in VEH and CIS‐treated cells. (I, J) Densitometric quantification of pro‐caspase‐3 (I) and cleaved‐caspase‐3 (J) in VEH and CIS‐treated cells. Whole western blot bands are reported in Figures S18–S23. Statistical analysis was performed using a two‐tailed unpaired student's *t*‐test (*n* = 3) (**p < 0.01 vs VEH). Data are presented as mean ± SEM. ASC, apoptosis‐associated speck‐like protein; C, cytoplasmic fraction; CIS, cisplatin; GAPDH, glyceraldehyde‐3‐phosphate dehydrogenase; GSDMD, gasdermin‐D; N, nuclear fraction; NF‐κB, nuclear factor kappa‐light‐chain‐enhancer of activated B‐cells; NLRP3, pyrin domain–containing‐3; VEH, vehicle.

Once the cisplatin model was set, we explored the possible alterations of molecular pathways typically associated with ototoxicity. To this end, we investigated the activation of NF‐κB, a transcription factor with a pivotal role in the regulation of pro‐inflammatory and survival processes [37]. Given that NF‐κB activation is denoted by its cytoplasm‐to‐nucleus translocation, we first examined NF‐κB localization in UB/OC1 cells upon cisplatin or vehicle treatment through immunofluorescence and confocal microscopy (Figure 3A). In vehicle‐treated cells, the NF‐κB signal (in red) was predominantly localized in the cytoplasm, whereas cisplatin markedly induced the accumulation of NF‐κB into the nucleus (Figure 3A). This observation was further supported by quantifying NF‐κB levels in cytosolic and nuclear protein fractions by Western blotting (Figure 3B–D). Densitometric analysis revealed that indeed the nuclear fractions of NF‐κB were significantly higher (*p* = 0.0097) in cisplatin‐treated versus vehicle‐treated cells (Figure 3C). Moreover, no differences were found in the cytoplasmic NF‐κB levels between cisplatin‐treated and vehicle‐treated cells (Figure 3D), suggesting that the nuclear accumulation of NF‐κB was likely due to both translocation and increased expression. On this basis, we proceeded to investigate downstream targets of NF‐κB activation, such as NLRP3 inflammasome complex and Caspase‐3 activation.

The NF‐κB/NLRP3 axis has been shown to mediate inflammatory processes, and notably to be involved in ototoxicity [38]. NLRP3, as well as the key components of the inflammasome complex ASC and GSDMD, were found to be expressed in UB/OC1 cells (Figure 3E–G). Yet, we failed to detect caspase‐1 and its cleaved form, the key downstream effector of inflammasome activation [38] (Figure S22), or its downstream substrates N‐GSDMD and IL‐1β (Figure S24), overall indicating that the inflammasome pathway was inactive in UB/OC1 cells. Furthermore, cisplatin did not induce inflammasome activation, since no significant changes in inflammasome proteins were observed compared to vehicle‐treated cells (Figures 3E–G and S20–S22). We concluded that the NF‐κB/NLRP3 axis was not activated by cisplatin, and that an alternative signaling pathway may be activated by NF‐κB under our experimental conditions. To this end, we investigated Caspase‐3 signaling activation, which is implicated in apoptosis [39] and has been reported in cisplatin‐induced auditory HC damage [40, 41]. Pro‐Caspase‐3 was unchanged between vehicle and cisplatin treated cells (Figure 3H,I), whereas its cleaved form was significantly up‐regulated in cisplatin treated cells (*p* = 0.0070) (Figure 3H,J), indicating the activation of apoptotic events in our ototoxicity model [39].

### 3.4 Cisplatin Downregulates the Expression of CB_2_R

**FIGURE 4:**
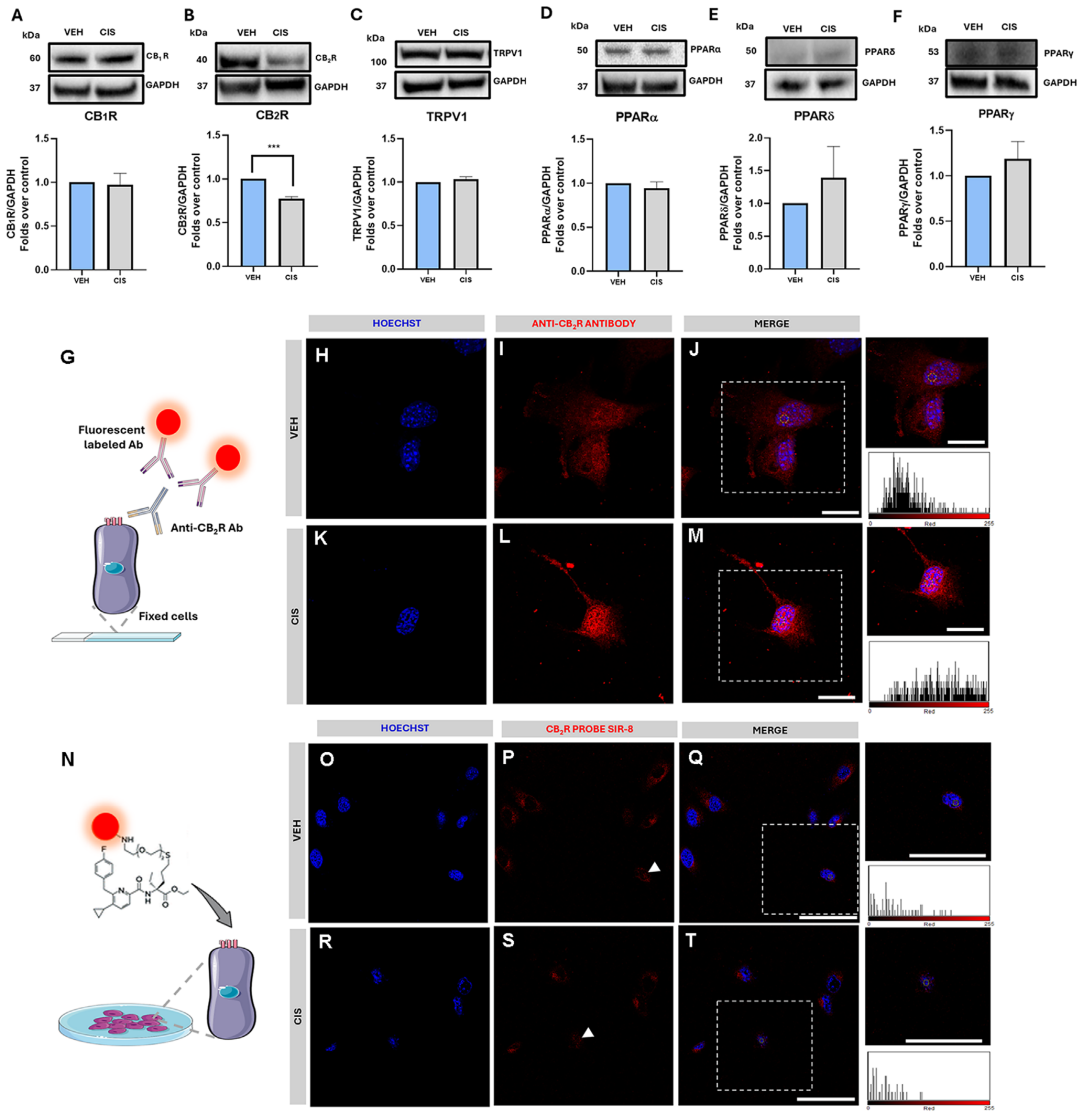
Analysis of eCB receptors in UB/OC1 cells upon cisplatin treatment. (A–F) Representative Western blot bands and densitometric quantification of CB_1_R (A), CB_2_R (B), TRPV1 (C), PPARα (D), PPARδ (E), and PPARγ (F) with respective GAPDH housekeeping in VEH and CIS‐treated cells. Data is presented as mean ± SEM. Statistical analysis was performed using a two‐tailed unpaired Student's *t*‐test (*n* = 3) (***p ≤ 0.001 vs VEH). Whole Western blot bands are reported in Figures S25–S30. (G) Schematic representation of the immunofluorescence staining method using anti‐CB_2_R antibody. (H–J) Representative confocal images of VEH‐treated cells immunostained with anti‐CB_2_R (red) and counterstained with Hoechst nuclear dye (blue); (K–M) Representative confocal images of CIS‐ treated cells immunostained with anti‐CB2R (red) and counterstained with Hoechst nuclear dye (blue). Scale bar: 20 μm. (N) Schematic representation of the CB_2_R selective probe SiR‐8 live‐cell staining. (O–Q) Representative confocal microscopy images of VEH‐treated cells stained with CB_2_R SiR‐8 probe (red) and counterstained with Hoechst nuclear dye (blue); (R–T) Representative confocal microscopy images of CIS‐treated cells stained with CB_2_R SiR‐8 probe (red) and counterstained with Hoechst nuclear dye (blue). Scale bar: 75 μm. White dotted boxes show zoomed‐in views of UB/OC1 cells, and the corresponding fluorescence intensity histograms are representative of the yellow circle‐marked areas. Live‐cell time‐lapse video is provided in the Supporting Information. CB_1_R, cannabinoid receptor 1; CB_2_R, cannabinoid receptor 2; CIS, cisplatin; eCBs, endocannabinoids; GAPDH, glyceraldehyde‐3‐phosphate dehydrogenase; PPAR α, γ, δ, peroxisome proliferator‐activated nuclear receptors α, γ, δ; TRPV1, transient receptor potential vanilloid receptor 1; VEH, vehicle.

We then explored the possible perturbation of the ECS by cisplatin in our ototoxicity model. Firstly, we investigated the primary eCBs‐binding receptors CB_1_R, CB_2_R, TRPV1, PPARα, δ, and γ. Interestingly, a significant downregulation of CB_2_R (*p* = 0.0006)—but not of any other ECS element—was observed in cisplatin‐treated versus vehicle‐treated cells (Figure 4A–F), suggesting a selective targeting of CB_2_R by cisplatin. Hence, we further investigated cisplatin‐induced CB_2_R alterations by looking into the receptor localization through two methodological approaches: (i) classical immunofluorescence staining on the basis of the use of anti‐CB_2_R antibody [42] (Figure 4G–M), and (ii) live‐cell imaging of CB_2_R by using the specific fluorescent agonist SiR‐8 [43] (Figure 4N–T). In addition to a heterogeneous cytoplasmic localization, CB_2_R was also observed in the nucleus of vehicle‐treated UB/OC1 cells through both imaging techniques. Moreover, cisplatin treatment failed to induce any significant difference in CB_2_R localization assessed by either immunofluorescence (Figure 4) or time‐lapse assays (see Videos S1 and S2).

### 3.5 Cisplatin Causes Selective Perturbation of 2‐AG Metabolism

**FIGURE 5:**
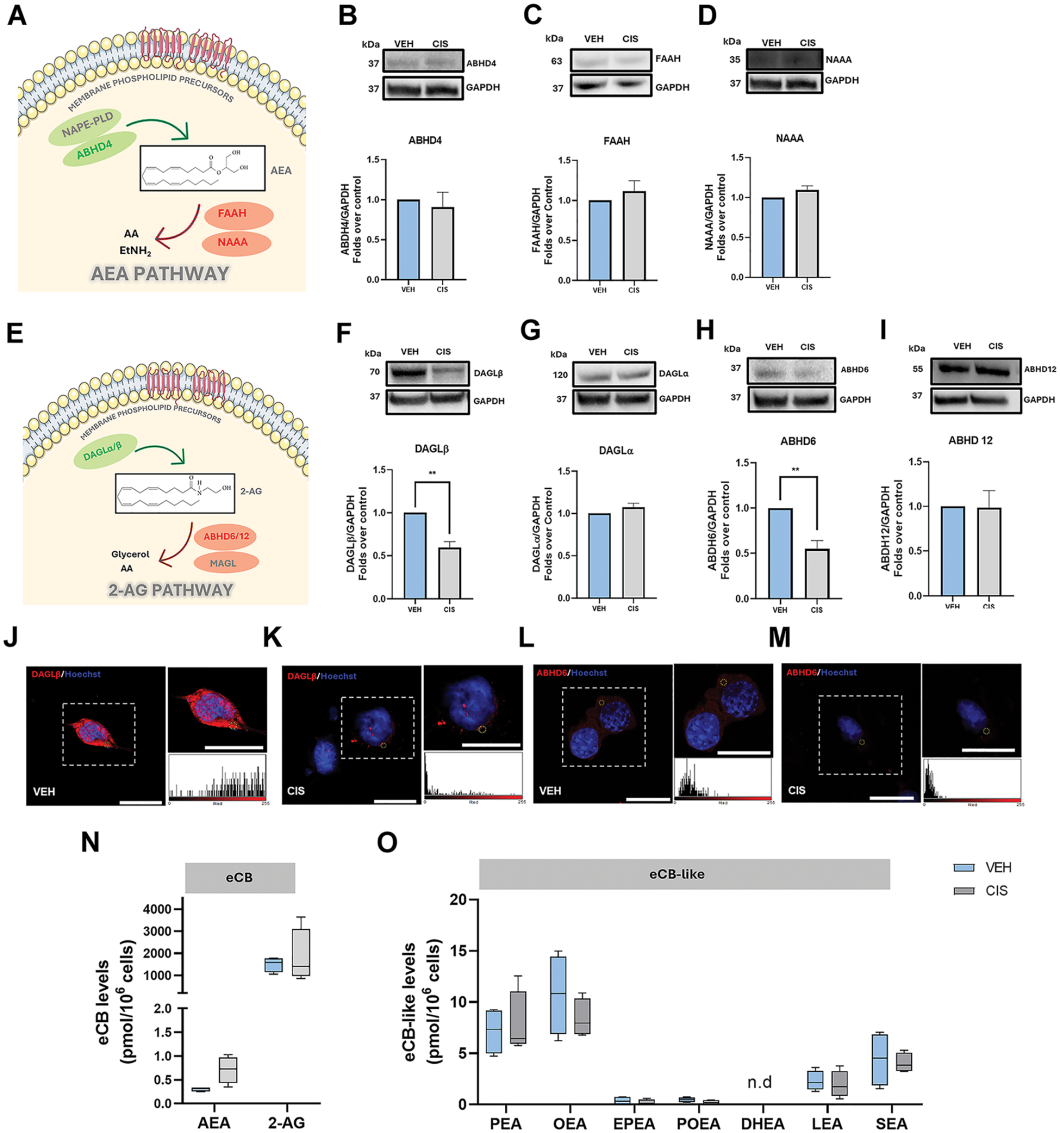
Effects of cisplatin on eCB metabolism. (A) Schematic representation of AEA metabolism. The biosynthetic enzymes are marked in green; the degradative enzymes are marked in red, and the enzymes that were not found to be expressed in the cell line are marked in gray. (B–D) Representative Western blots of ABHD4 (B), FAAH (C), NAAA (D), and corresponding densitometric quantitation with the GAPDH housekeeping in VEH and CIS‐treated cells. (E) Schematic representation of 2‐AG metabolism. The biosynthetic enzymes are marked in green; the degradative enzymes are marked in red, and the enzymes that were not expressed in the cell line are marked in gray. (F–I) Representative Western blots of DAGLβ (F), DAGLα (G), ABHD6 (H), ABHD12 (I), and corresponding densitometric quantitation with the GAPDH housekeeping in VEH and CIS‐treated cells. (J–M) Representative confocal microscopy images of DAGLβ (red) (J, K) and ABHD6 (red) (L, M) in VEH‐ and CIS‐treated cells, counterstained with Hoechst nuclear dye (blue); scale bar: 20 μm. White dotted boxes show zoomed‐in views of UB/OC1 cells, and the corresponding fluorescence intensity histograms are representative of the yellow circle‐marked areas. Data is presented as mean ± SEM Statistical analysis was performed using two‐tailed unpaired Student's *t*‐test (*n* = 3) (**p ≤ 0.01 vs VEH). Whole Western blot bands are reported in Figures S31–S39. (N) Levels of AEA and 2‐AG measured using UHPLC–MS/MS in VEH‐ and CIS‐treated UB/OC1cells. (O) Levels of eCB‐like compounds PEA, OEA, POEA, EPEA, DHEA, LEA and SEA, in VEH‐ and CIS‐treated UB/OC1 cells measured using UHPLC–MS/MS. Data are represented as a box plot with whisker plots ranging from min to max values for 4 independent experiments. When the expression of the lipids fell below the lower limit of quantification (LOQ), it was represented as 0. Statistical analysis was performed using two‐way ANOVA with Bonferroni *post hoc* comparison (*n* = 4). The data is presented as a box plot with whiskers ranging from min to max values. 2‐AG, 2‐arachidonoylglycerol; AA, arachidonic acid; ABHD 4, α/β hydrolase domain‐containing protein 4; ABHD 6/12, α/β hydrolase domain‐containing protein 6/12; AEA, *N*‐arachidonoylethanolamine; CIS, cisplatin; DAG, diacylglycerol; DAGLα/β, diacylglycerol lipases α and β; DHEA, docosahexaenoylethanolamine; EPEA, epoxyeicosatetraenoyl ethanolamide; EtNH_2_, ethanolamine; FAAH, fatty acid amide hydrolase; GAPDH, glyceraldehyde‐3‐phosphate dehydrogenase; LEA, linoleoylethanolamide; MAGL, monoacylglycerol lipase; NAPE‐PLD, *N*‐acyl‐phosphatidylethanolamines‐specific phospholipase D; NArPE, *N*‐arachidonoyl‐phosphatidylethanolamine; OEA, *N*‐oleoylethanolamine; PEA, *N*‐palmitoylethanolamine; POEA, *N*‐palmitoleoylethanolamine; SEA, *N*‐stearoylethanolamine; VEH, vehicle.

We then explored the impact of cisplatin on AEA and 2‐AG metabolic enzymes in UB/OC1 cells. As for AEA (Figure 5A), we did not find any significant changes between groups in the expression levels of its biosynthetic enzyme ABHD4 (Figure 5B), nor of its degradative enzymes FAAH (Figure 5C) and NAAA (Figure 5D). Instead, cisplatin perturbed 2‐AG metabolism, schematically reported in Figure 5E. Indeed, it caused a significant downregulation of the 2‐AG biosynthetic enzyme DAGLβ (Figure 5F, *p* = 0.0041), but not DAGLα (Figure 5G), as well as of the 2‐AG degradative enzyme ABHD6 (Figure 5H, *p* = 0.0079), but not ABHD12 (Figure 5I). These data were also confirmed by immunofluorescence staining on cisplatin‐treated and vehicle‐treated cells (Figure 5J–M). Taken together, under our experimental conditions, cisplatin selectively targeted the 2‐AG metabolic pathway without affecting that of AEA.

### 3.6 Cisplatin Does Not Alter Endogenous Tone of eCBs and Congeners in UB/OC1 Cells

On the basis of these results, we hypothesized that eCB levels might be altered by cisplatin, particularly in the case of 2‐AG. However, no changes were observed between groups for either the major eCBs AEA and 2‐AG (Figure 5N) or other eCB‐like compounds like PEA, LEA, OEA, SEA, POEA, and EPEA (Figure 5O).

### 3.7 Cisplatin Reduces CB_2_R, DAGLβ and ABHD6 in the Murine OC

**FIGURE 6:**
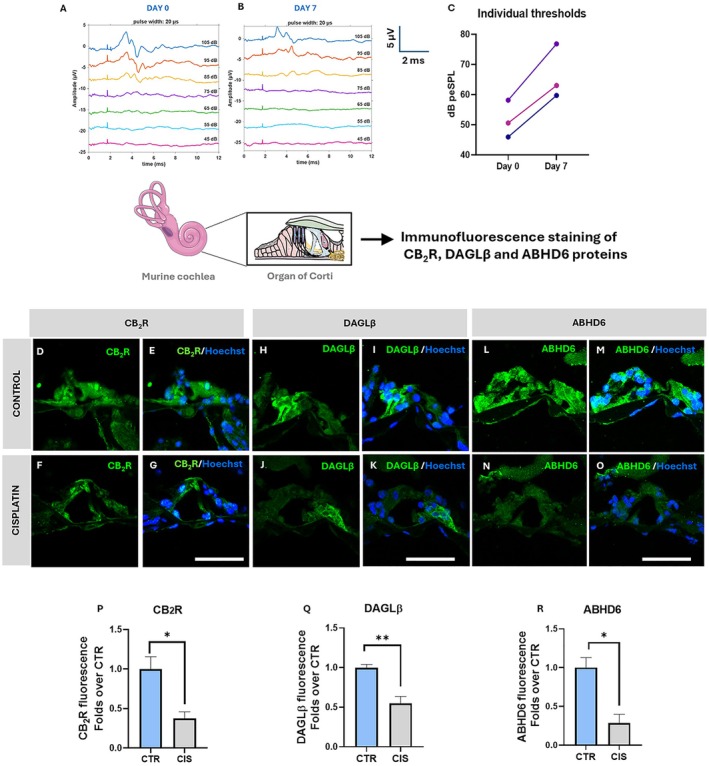
ABR analysis in the in vivo ototoxic model and Immunofluorescence of CB_2_R, DAGLβ and ABHD6 in the mouse OC upon cisplatin‐induced ototoxicity. (A, B) Representative ABR waveform of the group on day 0 (A) and on day 7 (B). (C) Individual thresholds of 3 animals (each color represents an animal) on day 0 and day 7 shown as symbols with connecting lines between days 0 and 7. (D–G) Representative confocal microscopy images of CB_2_R (green) counterstained with Hoechst nuclear dye (blue) in control (D, E) and cisplatin‐treated OC (F, G). (H–K) Representative confocal microscopy images of DAGLβ (green) counterstained with Hoechst nuclear dye (blue) in control (H, I) and cisplatin‐treated OC (J, K). (L–O) Representative confocal microscopy images of ABHD6 (green) counterstained with Hoechst nuclear dye (blue) in control (L, M) and cisplatin‐treated OC (N, O). (P–R) Fluorescence intensity of CB_2_R (P), DAGLβ (Q) and ABHD6 (R) in CTR and CIS‐treated OC, scale bar‐50 μm. Statistical analysis is performed using two‐tailed unpaired student's *t*‐test (*p < 0.05 vs CTR, **p < 0.01 vs CTR). Data is presented as mean ± SEM (*n* = 3). Appropriate negative controls for IHC are provided in Figure S42. ABHD6, α/β hydrolase domain‐containing protein; CB_2_R, cannabinoid receptor 2; CIS, cisplatin; DAGLβ, diacylglycerol lipase β; IF, immunofluorescence.

To enhance the translational significance of our findings, we moved to in vivo experiments by establishing a mouse model of cisplatin‐induced ototoxic damage (Figure S1). Our model was developed on the basis of published protocols [26], and exhibited functional ototoxic damage in three animals, which were therefore included in the study. Specifically, the ABR analysis revealed significant functional impairment (> 10 dB threshold shifts) upon cisplatin on day 7 compared to day 0, as seen from the representative waveforms of the groups (Figure 6A,B) and the individual thresholds of the animals (Figure 6C). Importantly, the weights of the animals remained constant throughout the study, and no systemic toxicity was noted (Figure S41). In such a model, we performed IHC on basal OC cryosections to investigate the possible alterations of those ECS elements (i.e., CB_2_R, DAGLβ, and ABHD6) that were found to be altered in vitro by cisplatin. Of note, consistent with the in vitro data, we observed a significant decrease in the expression of CB_2_R (0.4‐fold over control; *p* = 0.0240) (Figure 6D–G,P) in cisplatin‐treated versus control animals, and we also confirmed CB_2_R nuclear localization under both conditions. Moreover, the 2‐AG metabolic enzymes DAGL β (0.5‐fold over control; *p* = 0.0099) (Figure 6H–K,Q) and ABHD6 (0.3‐fold over control; *p* = 0.0141) (Figure 11L–O,R) were significantly reduced in cisplatin‐treated animals compared with controls, overall confirming the in vitro data.

### 3.8 CB_2_R Antagonism Mitigates Cisplatin‐Induced Cell Death by Inhibiting Caspase‐3 Activation

**FIGURE 7:**
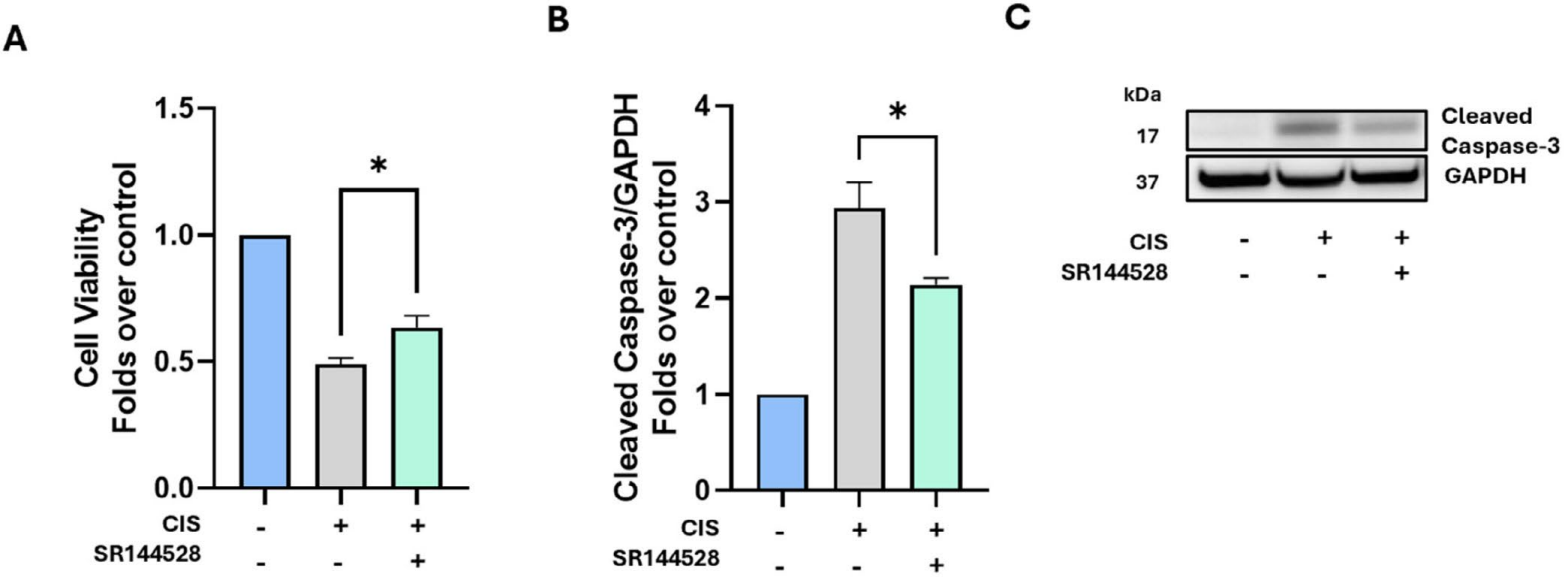
Pharmacological manipulation of CB_2_R in cisplatin‐treated cells. (A) Cell viability of UB/OC1 cells upon treatment with SR144528 (B), determined by MTT assay. Statistical analysis was performed using a two‐tailed unpaired student's *t*‐test (*n* = 6) (*p ≤ 0.05 vs CIS). Data are presented as mean ± SEM. (B, C) Densitometric quantitation of cleaved Caspase‐3 with the GAPDH housekeeping (B), with representative Western blots of all experimental groups (C). Statistical analysis was performed using a two‐tailed unpaired Student's *t*‐test between CIS and CIS + SR144528 (*n* = 3) (*p ≤ 0.05). Whole Western blot bands are reported in Figure S40. CB_2_R, cannabinoid receptor 2; CIS, cisplatin.

Once the ECS alterations observed in vitro were confirmed also in vivo, we sought to elucidate their functional implications. To this end, we focused on CB_2_R because of the availability of well‐established and selective antagonists [44, 45, 46], which allows for precise functional analysis. Pharmacological manipulation of CB_2_R was performed in vitro using the CB_2_R antagonist SR144528 (2 μM), in the presence of cisplatin. First, we confirmed that the selected concentration of the antagonist was not cytotoxic for UB/OC1 cells (Figure S43). Subsequently, MTT assay showed that combined treatment of cisplatin and SR144528 protected cells against cisplatin toxicity (*p* = 0.0256) (Figure 7A). On this basis, we hypothesized that this protective effect could be associated with the modulation of Caspase‐3, which was activated by cisplatin and is known to trigger apoptosis (Figure 3H–J). Consistent with this hypothesis, SR144528 reduced the activation of Caspase‐3, as indicated by decreased protein levels of its cleaved form compared to cells treated with cisplatin alone (Figure 7B,C). Collectively, these data demonstrate that blockade of CB_2_R mitigates cisplatin‐induced HC damage by inhibiting Caspase‐3 activation.

## 4 Discussion

Cisplatin‐induced ototoxicity is a major adverse effect of chemotherapy that profoundly impacts patients' quality of life. Effective management of these side effects critically depends on a thorough understanding of cisplatin's mechanisms of action within the auditory system, which remain largely unknown. In this study, we provide new insights into the molecular mechanisms triggered by cisplatin in auditory HCs, identifying selective components of the ECS as molecular targets of the drug in vitro and in vivo. These findings were grounded upon a systematic characterization of the complex ECS network in suitable auditory HC‐like cells, providing the first comprehensive molecular profiling of eCBs, their binding receptors, metabolic enzymes, and congeners. Below, we detail the main findings of the present study.

### 4.1 Identification of the ECS in Auditory HC‐Like UB/OC1 Cells

To date, the expression and function of the ECS in auditory HCs have been poorly investigated, and the available studies are generally focused on some of the eCBs‐binding receptors only [8, 9, 10, 11, 47]. Indeed, data on eCB metabolism is limited to one transcriptomic study [8], whereas no information is currently available on endogenous levels of eCBs and eCB‐like compounds within auditory models. To fill this knowledge gap, in the present study we performed a systematic molecular profiling of the main ECS elements in UB/OC1 cells, exploring the expression of primary receptor targets, classical and alternative eCB metabolic enzymes, and endogenous contents of these bioactive lipids. The UB/OC1 cells represent a suitable auditory HC‐like model because of the expression of key sensory HC markers, like Myo7a [36] that was also detected in the present investigation (Figure 2). In our cell model, we found the expression of primary eCBs‐binding receptors (CB_1_R, CB_2_R, TRPV1, and PPARα, δ and γ) (Figure 1), which extends literature data on the whole OC in animal models [12, 18, 47, 48, 49, 50, 51]. In addition, we provide new insights into CB_2_R sub‐cellular localization in HCs, demonstrating its presence in the nuclei of UB/OC1 cells (Figure 4). Such a nuclear localization of CB_2_R has also been observed in other cell lines [21, 52, 53, 54], but its functional implications remain as yet unknown, and the relevance of nuclear CB_2_R in cisplatin ototoxicity appears too speculative to support any conclusive statements.

In addition to eCBs‐binding receptors, we profiled the protein expression of eCB metabolic enzymes in UB/OC1 cells, showing that metabolic enzymes of both AEA (i.e., ABHD4, FAAH, and NAAA) and 2‐AG (i.e., DAGLα and β, ABDH6/12) are present in these cells (Figure 1). This observation suggests that AEA and 2‐AG metabolism in HCs engages alternative enzymes rather than the most common ones (i.e., ABHD4 instead of NAPE‐PLD for AEA biosynthesis; ABHD6 and ABHD12 instead of MAGL for 2‐AG degradation), pointing to the ABHD class of enzymes as key ECS components in HCs. Furthermore, we demonstrated that endogenous eCBs are indeed present in UB/OC1 cells, with 2‐AG being the most abundant compound (Figure 1). The latter finding aligns well with previous observations in the brain, where 2‐AG levels have been found to be ~170‐fold higher than AEA [55].

Overall, the presence of a full ECS in UB/OC1 cells supports the importance of eCB signaling in auditory function and lays the groundwork for future investigations into its contribution to hearing health and disease. Moreover, the discovery of eCBs in auditory HC‐like cells, along with the expression of their metabolic enzymes, adds new information to the understanding of ECS activity, indicating local synthesis and metabolism that may influence auditory function and thus offer new therapeutic targets for treating hearing impairment.

### 4.2 Cisplatin Modulates Distinct ECS Elements in OC Cells Both In Vitro and In Vivo

The next aim of the present study was to unveil the impact of cisplatin on the ECS by developing a suitable in vitro model of ototoxicity. Our model was characterized by cytotoxicity (Figure 2), as well as by other key molecular events typically involved in cisplatin‐induced ototoxicity, such as the reduction of the HC marker Myo7a (Figure 2), activation of NF‐κB and Caspase‐3 (Figure 3). In fact, the expression of Myo7a is known to be decreased by cisplatin because of the degeneration of HCs in in vivo models [34, 35]. Furthermore, activation of NF‐κB and Caspase‐3 shown here extends previous data reported elsewhere in both in vitro and in vivo ototoxicity models [39, 40, 41, 56]. Under our experimental conditions, we found distinct alterations of the ECS by cisplatin, including downregulation of CB_2_R protein levels. Notably, we show that CB_2_R antagonism using SR144528 protects UB/OC1 cells from cisplatin‐induced cell damage by inhibiting Caspase‐3 activation (Figure 7). This is consistent with the pro‐apoptotic role of CB_2_R observed in other cellular contexts, like cancer [57, 58]. Furthermore, this finding seems relevant because it provides new mechanistic insights into the role of CB_2_R in cisplatin‐induced ototoxicity, indicating that receptor antagonism may serve as a new therapeutic strategy for the management of cisplatin‐induced hearing loss. Additionally, here we show that cisplatin‐induced CB_2_R alterations in HCs are also associated with the imbalance of key metabolic enzymes of eCBs, namely, DAGLβ and ABHD6, which are known to be involved in the biosynthesis and inactivation of 2‐AG, respectively (Figures 5 and 6). Of note, a similar scenario has been documented in other organs upon treatment with cisplatin, such as the brain and the gastrointestinal system [59]. Nonetheless, endogenous levels of eCBs (in particular 2‐AG) remained unaffected by cisplatin (Figure 5), which can be likely due to the concomitant reduction of both the biosynthetic (DAGLβ) and the degradative enzyme (ABHD6) of 2‐AG. In line with this hypothesis, the ratio of DAGLβ/ABHD6 protein levels was found to be similar in vehicle‐treated and cisplatin‐treated cells (Figure S44). Enzyme activity assays would also be helpful to fully interpret these findings. However, assays for ABHD6 are currently available for cells transiently overexpressing the enzyme [60]; assessing only DAGLβ activity would not allow a reliable or interpretable indication of 2‐AG turnover, since both enzymes were similarly modulated by cisplatin in our study. In the future, the development of effective ABHD6 activity assays will be crucial to fully elucidate the contribution of these enzymes to eCB metabolism in ototoxicity. At any rate, it should be noted that DAGLβ and ABHD6 control a number of processes in the human body that do not require a change in 2‐AG levels, from the regulation of signaling pathways to metabolism of structural lipids and synaptic transmission [61]. Therefore, it is plausible that alternative ototoxic mechanisms may be mediated by cisplatin through DAGLβ and ABHD6, without affecting 2‐AG levels. For instance, DAGLβ was recently shown to hydrolyze triacylglycerols (TAGs) containing polyunsaturated fatty acids (PUFA) in macrophages, indicating a role that extends beyond the classical eCB route [62]. On the other hand, ABHD12 was originally known for its involvement in polyneuropathy, hearing loss, ataxia, retinosis pigmentosa, and cataract (PHARC) syndrome [63], but then it was found to regulate brain lysophosphatidylserine (LPS) levels in an eCB‐independent manner, thus resulting in neurobehavioral abnormalities [64]. It should be also reminded that, whereas conventional mass spectrometric methods provide valuable information on overall eCB levels, they do not reveal spatiotemporal or subcellular eCB turnover. This limitation may hide more subtle and dynamic eCB changes, which could potentially be induced by cisplatin in auditory HCs. In the future, this gap could be filled by recently developed—and not yet commercially available—technologies on the basis of genetically encoded eCB sensors, which enable real‐time monitoring of eCB signaling even at the subcellular level [65, 66].

Overall, the present investigation highlights distinct ECS elements as new players for therapeutic intervention, laying the foundation for further studies to investigate the role of this complex lipid network in cisplatin‐induced ototoxicity.

## Author Contributions

Conceptualization: A.T. and M.M.; methodology: S.P., A.T., C.D.M., C.U., G.E.F., F.D.V., and F.F.; data curation: S.P.; formal analysis: S.P.; writing – original draft: S.P. and A.T.; writing – review and editing: S.P., A.T., C.D.M., F.D.V., F.F., D.C., M.N., H.V., A.A., M.A., and M.M.; funding acquisition: M.M.; supervision: A.T., H.V., and M.M.

## Funding

This investigation is part of Sakthimala Palaniappan's PhD program, which is supported by the Italian Ministry of Research (MUR) and Dompé Farmaceutici S.p.A under the competitive grant “Innovative PhD with Industrial Characterization‐PNRR‐2022/2023 Ministerial Decree n.352 of 9 April 2022‐CUP: E11I22000230001”, through a collaborative research agreement with the University of L'Aquila supervised by MM. The funders had no role in the design of the study, nor in the collection, analyses, or interpretation of data.

## Conflicts of Interest

The authors declare no conflicts of interest.

## Supporting information


**Data S1:** Supporting Information.

**Video S1:** SiR‐8 live‐cell imaging clip of cisplatin‐treated UB/OC1 cells.

**Video S2:** SiR‐8 live‐cell imaging clip of vehicle‐treated UB/OC1 cells.

## Data Availability

The data that support the findings of this study are available in the Materials and Methods, Results, and/or Supporting Information of this article.
