# Supplementary material for: Unraveling Endocannabinoid Signaling Pathways in Cisplatin‐Induced Ototoxicity
Source: FASEB J. 2026 Feb 17;40(4):e71568. doi: 10.1096/fj.202502888RR (PMC12911942; doi:10.1096/fj.202502888RR)
Supplement: Supplementary file 1 — Data S1: Supporting Information. [file FSB2-40-e71568-s003.docx]

**Unraveling Endocannabinoid Signaling Pathways in Cisplatin-induced Ototoxicity**

**Authors**

Sakthimala Palaniappan ^1,#^, Annamaria Tisi ^1,#^, Camilla Di Meo ^1^, Cristina Urbano ^2^, Georgina E Fenton ^3,4^ Francesco Della Valle ^5^, Federico Fanti ^5^, Dario Compagnone ^5^, Marc Nazarè ^6^, Huib Versnel ^3,4^, Andrea Aramini ^7,§^, Marcello Allegretti^7,§^, Mauro Maccarrone ^1,2,§,^*

^#^Joint first authorship, ^§^Joint senior authors

*Corresponding author

Department of Biotechnological and Applied Clinical Sciences, University of L'Aquila, Via Vetoio snc, 67100 L’Aquila, Italy

Tel. (+39) 0862-433547

[mauro.maccarrone@univaq.it](mailto:mauro.maccarrone@univaq.it)

**Affiliations**

^1^ Department of Biotechnological and Applied Clinical Sciences, University of L'Aquila, 67100 L'Aquila, Italy.

^2^ European Center for Brain Research (CERC), Santa Lucia Foundation IRCCS, Rome, Italy.

^3^ Department of Otorhinolaryngology and Head & Neck Surgery, University Medical Center Utrecht, Utrecht University, Utrecht, the Netherlands

^4^ UMC Utrecht Brain Center, Utrecht University, Utrecht, the Netherlands

**^5^ ^[[1]](#footnote-1)^**Department of Bioscience and Technology for Food, Agriculture and Environment, University of Teramo, Teramo, Italy.

^6^ Leibniz Research Institute for Molecular Pharmacology (FMP), Campus Berlin-Buch, 13125 Berlin, Germany

^7^ Dompé Farmaceutici Spa, via Campo di Pile, 1, L'Aquila, Italy.

**Supplementary Figure S1. Schematic representation of the *in vivo* experimental design to induce cisplatin-induced ototoxicity in mice.** Mice underwent the deafening procedure by systemic administration of cisplatin and furosemide; click evoked ABR was measured on day 0 and day 7 to assess auditory function. Abbreviations: ABR: Auditory brainstem response; CIS: cisplatin; FUR: Furosemide; IP: intraperitoneal.

**
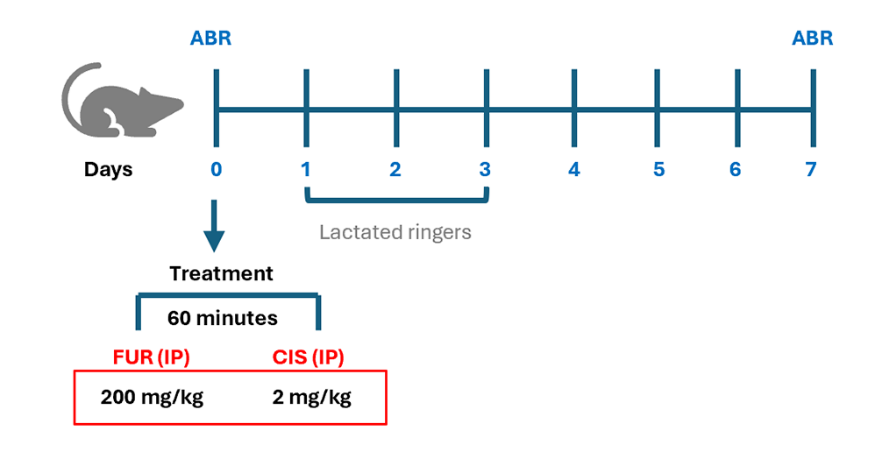
**

**Supplementary Figure S2**. **Western blot images of CB_1_R with its respective positive control.** The images show original Western blot bands of CB_1_R in mouse brain tissue lysate (B) and UB/OC1 cells (UB/OC1) with respective GAPDH housekeeping.


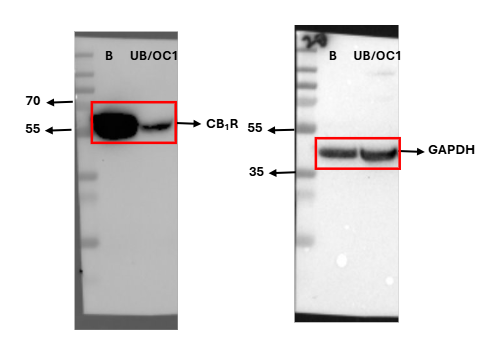


**Supplementary Figure S3**. **Western blot images of CB_2_R with its respective positive control.** The images show original Western blot bands of CB_2_ in mouse liver tissue lysate (L) and UB/OC1 cells (UB/OC1) with respective GAPDH housekeeping.


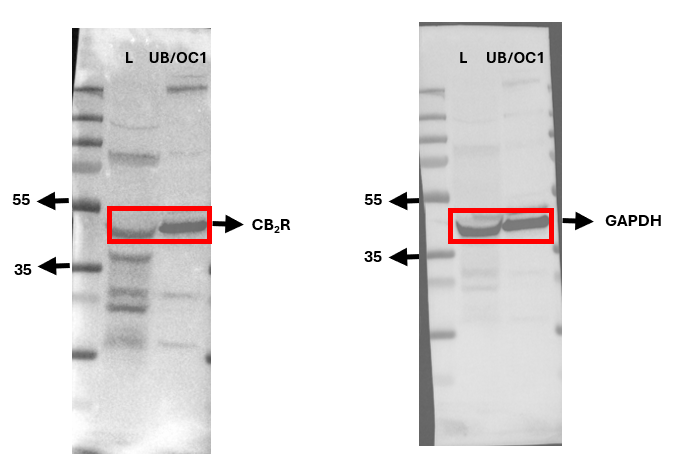


**Supplementary Figure S4**. **Western blot images of TRPV 1 with its respective positive control.** The images show original Western blot bands of TRPV 1 in mouse brain tissue lysate (B) and UB/OC1 cells (UB/OC1) with respective GAPDH housekeeping.

**
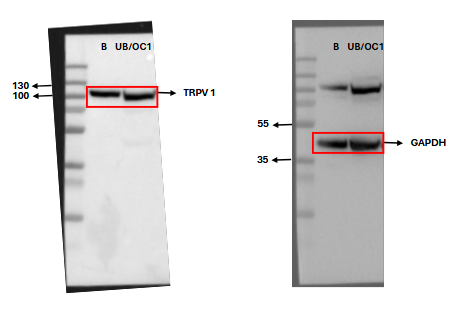
**

**Supplementary Figure S5**. **Western blot images of PPAR α with its respective positive control.** The images show original Western blot bands of PPAR α in UB/OC1 (UB/OC1) cells and mouse heart tissue lysate (H) with respective GAPDH housekeeping.


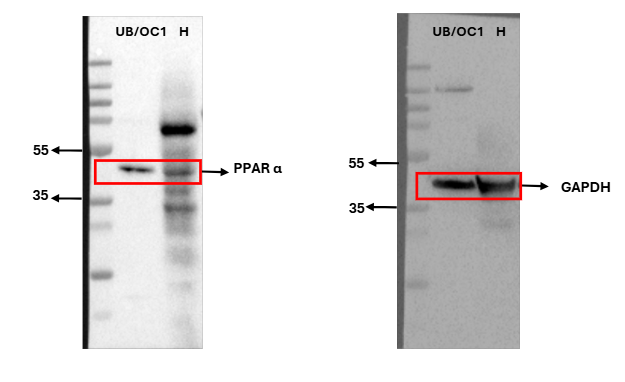


**Supplementary Figure S6**. **Western blot images of PPAR δ with its respective positive control.** The images show original Western blot bands of PPAR δ in mouse brain tissue lysate (B) and UB/OC1 cells (UB/OC1) with respective GAPDH housekeeping.


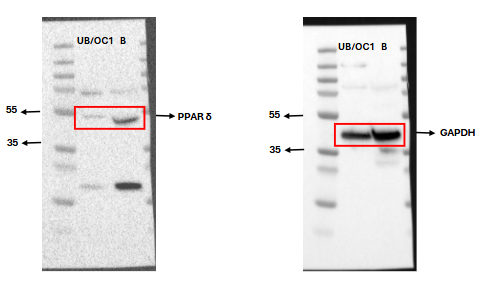


**Supplementary Figure S7**. **Western blot images of PPAR γ with its respective positive control.** The images show original Western blot bands of PPAR γ in UB/OC1 (UB/OC1) cells and mouse heart tissue lysate (H) and with respective GAPDH housekeeping.


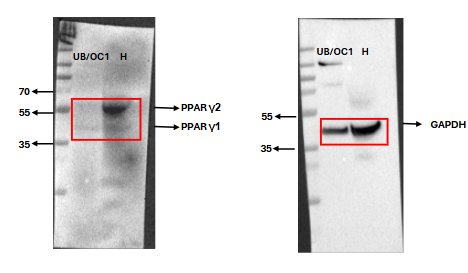


**Supplementary Figure S8**. **Western blot images of NAPE-PLD.** The images show original Western blot bands of NAPE-PLD in mouse brain tissue lysate (B) and UB/OC1 cells (UB/OC1) with respective GAPDH housekeeping

**
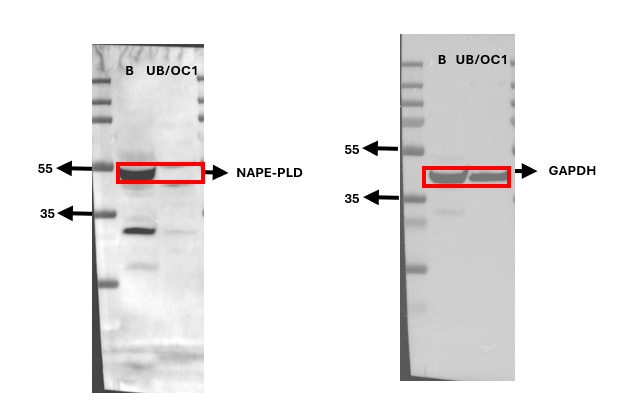
**

**Supplementary Figure S9**. **Western blot images of ABHD 4.** The images show original Western blot bands of ABHD 4 in UB/OC1 cells (UB/OC1) and mouse brain tissue lysate (B) with respective GAPDH housekeeping.


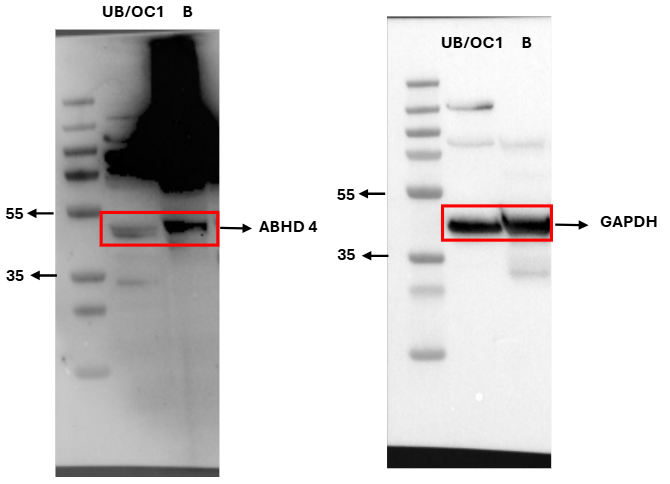


**Supplementary Figure S10. Western blot images of FAAH.** The images show original Western blot bands of FAAH in UB/OC1 cells (UB/OC1) and mouse brain tissue lysate (B) with respective GAPDH housekeeping.


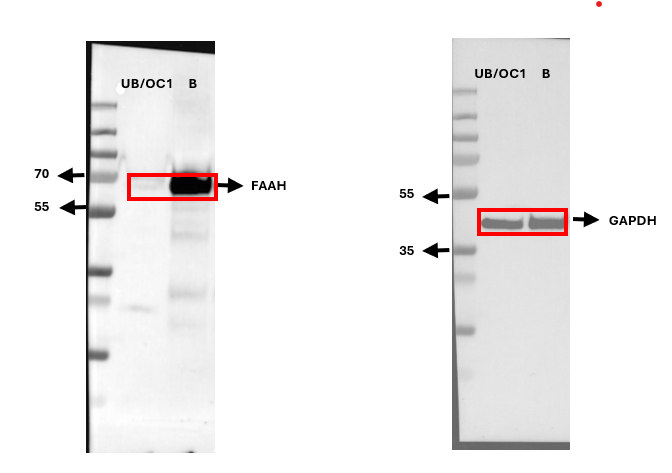


**Supplementary Figure S11.** **Western blot images of NAAA.** The images show original Western blot bands of NAAA in UB/OC1 cells (UB/OC1) and mouse brain tissue lysate (B) with respective GAPDH housekeeping.

**
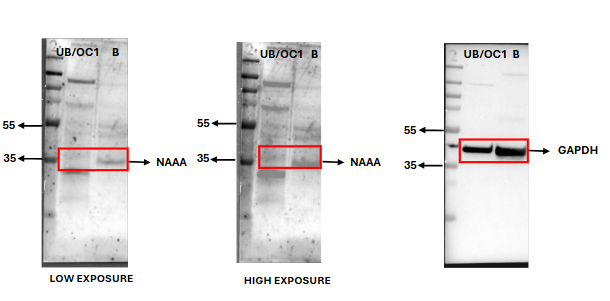
**

**Supplementary Figure S12**. **Western blot images of DAGL α with its respective positive control.** The images show original Western blot bands of DAGL α in mouse brain tissue lysate (B) and UB/OC1 cells (UB/OC1) with respective GAPDH housekeeping.


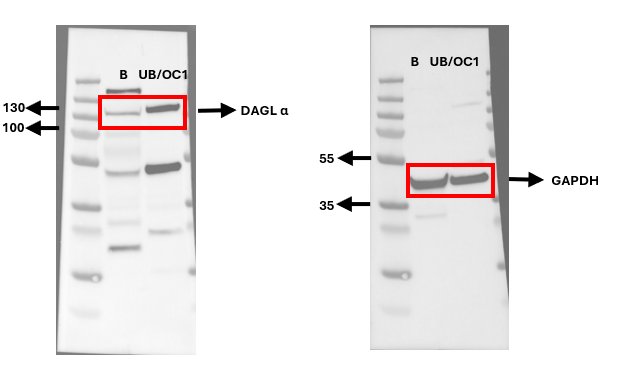


**Supplementary Figure S13**. **Western blot images of DAGL β with its respective positive control.** The images show original Western blot bands of DAGL β in mouse brain tissue lysate (B) and UB/OC1 cells (UB/OC1) with respective GAPDH housekeeping.


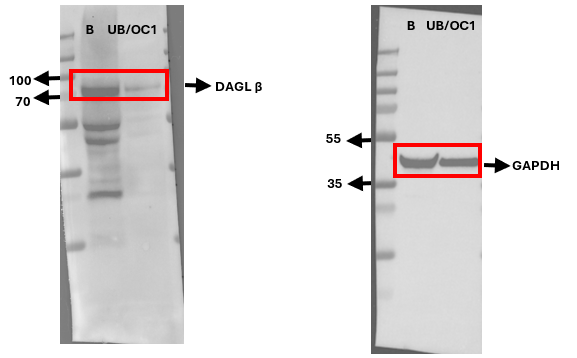


**Supplementary Figure S14**. **Western blot images of MAGL.** The images show original Western blot bands of MAGL in mouse brain tissue lysate (B) and UB/OC1 cells (UB/OC1) with respective GAPDH housekeeping.

**
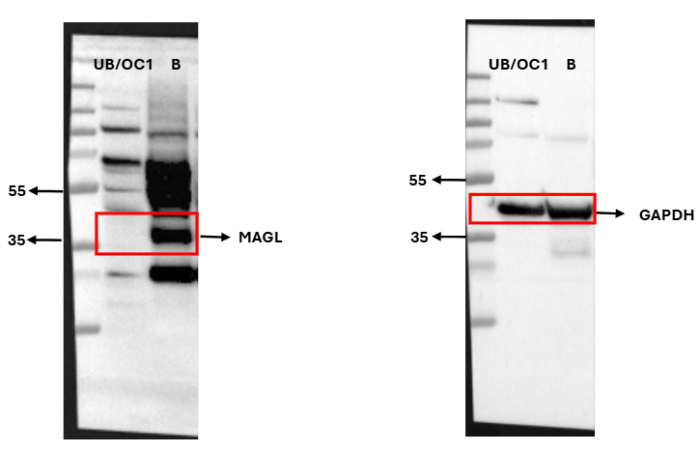
**

**Supplementary Figure S15**. **Western blot images of ABHD 6** **with its respective positive control.** The images show original Western blot bands of ABHD 6 in mouse brain tissue lysate (B) and UB/OC1 cells (UB/OC1) with respective GAPDH housekeeping.


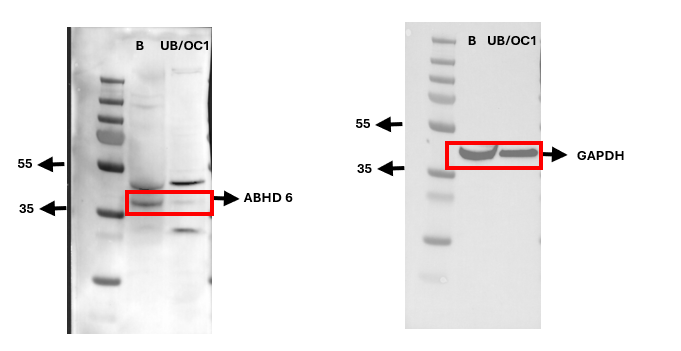


**Supplementary Figure S16**. **Western blot images of ABHD 12** **with its respective positive control.** The images show original Western blot bands of ABHD12 in mouse brain tissue lysate (B) and UB/OC1 cells (UB/OC1) with respective GAPDH housekeeping.


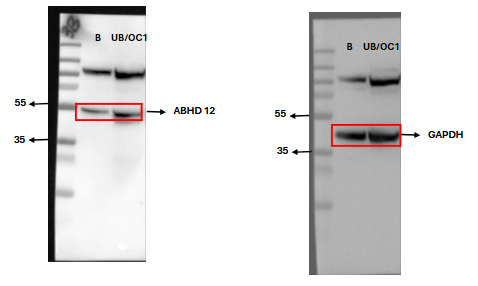


**Supplementary Figure S17. Whole** **Western blot images of Myosin 7a.** The images show original Western blot bands of Myosin 7a in vehicle treated cells (V) and cisplatin treated cells (T) with respective GAPDH housekeeping.


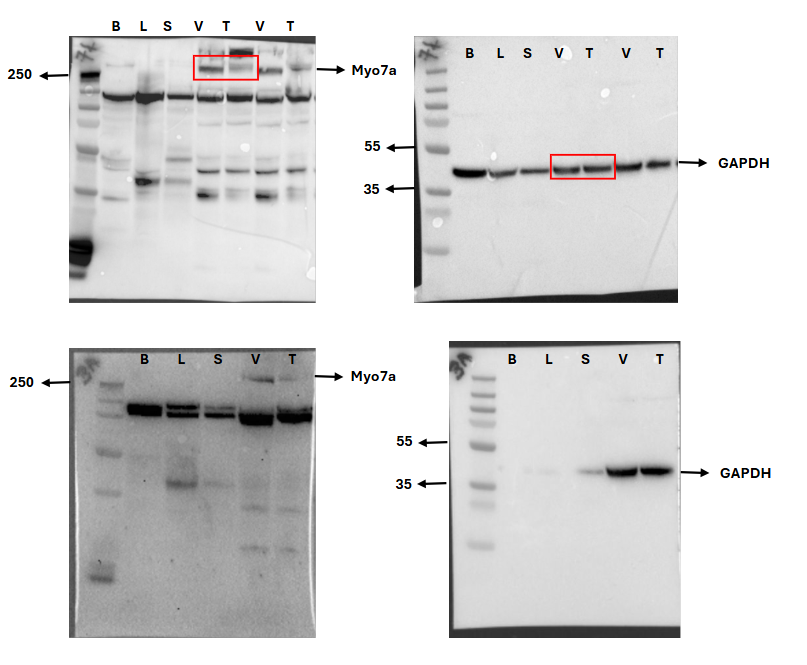


**Supplementary Figure S18.** **Whole** **Western blot images of NF-κB** **.** The images show original Western blot bands of NF-κB in vehicle treated cells (V) and cisplatin treated cells (T) in cytoplasmic (C) and nuclear (N) compartments along with the cytoplasmic marker αβ tubulin and nuclear marker Lamin A/C.


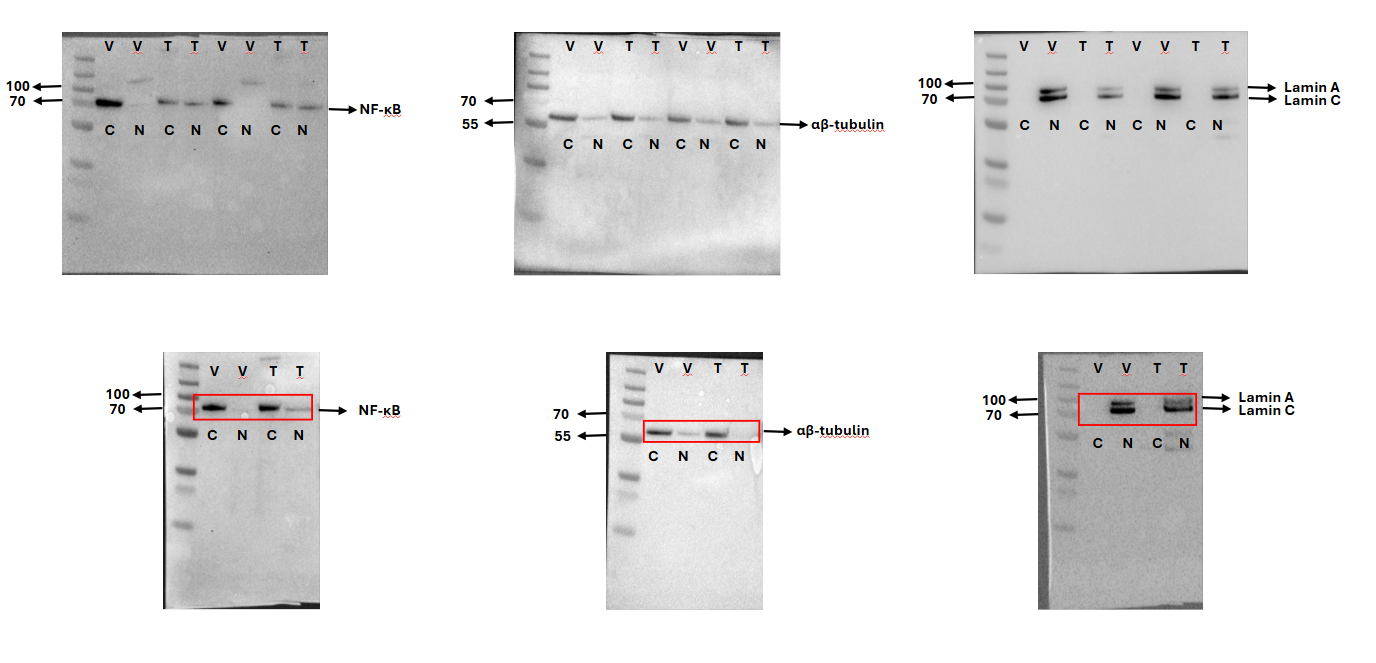


**Supplementary Figure S19**. **Whole** **Western blot images of NLRP3.** The images show original Western blot bands of NLRP3 in vehicle treated cells (V) and cisplatin treated cells (T) with respective GAPDH housekeeping.


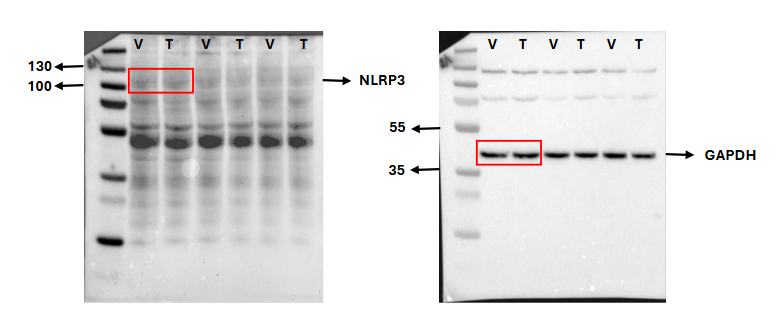


**Supplementary Figure S20**. **Whole** **Western blot images of ASC.** The images show original Western blot bands of ASC in vehicle treated cells (V) and cisplatin treated cells (T) with respective GAPDH housekeeping.


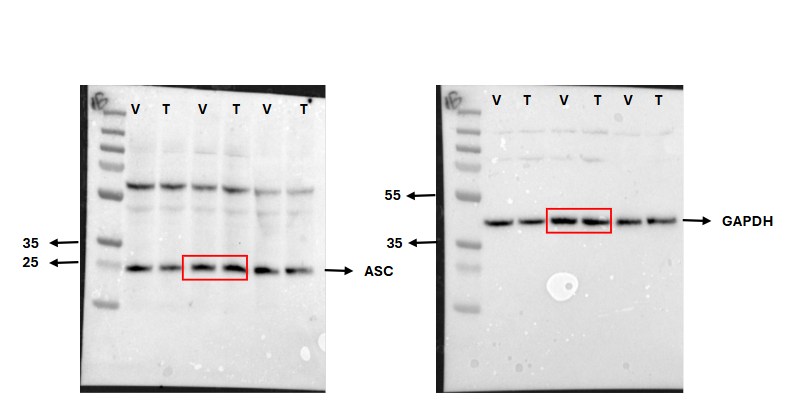


**Supplementary Figure S21**. **Whole** **Western blot images of GSDMD.** The images show original Western blot bands of GSDMD and N-GSDMD in vehicle treated cells (V) and cisplatin treated cells (T) with respective GAPDH housekeeping.


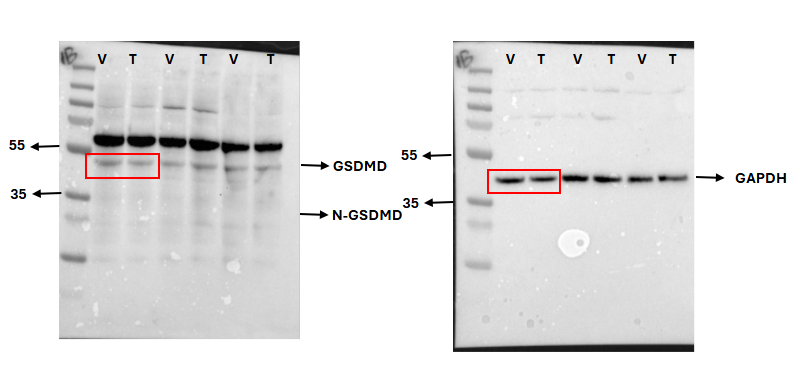


**Supplementary Figure S22**. **Whole** **Western blot images of Caspase-1.** The images show original Western blot bands of Pro-Caspase-1 and cleaved-caspase-1 in vehicle treated cells (V) and cisplatin treated cells (T) with respective GAPDH housekeeping.


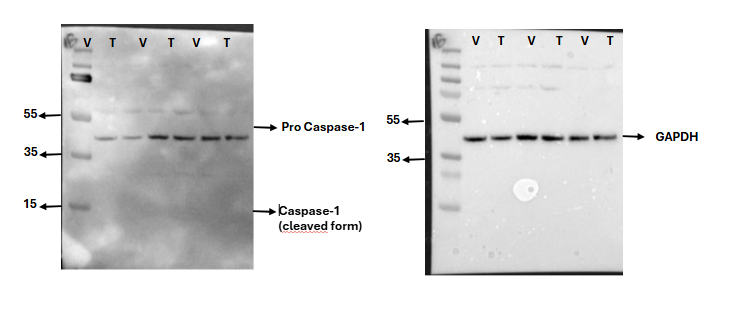


**Supplementary Figure S23. Whole** **Western blot images of Pro-Caspase-3 and cleaved Caspase-3.** The images show original Western blot bands of Pro-Caspase-3 and cleaved Caspase-3 in vehicle treated cells (V) and cisplatin treated cells (T) with respective GAPDH housekeeping.

**
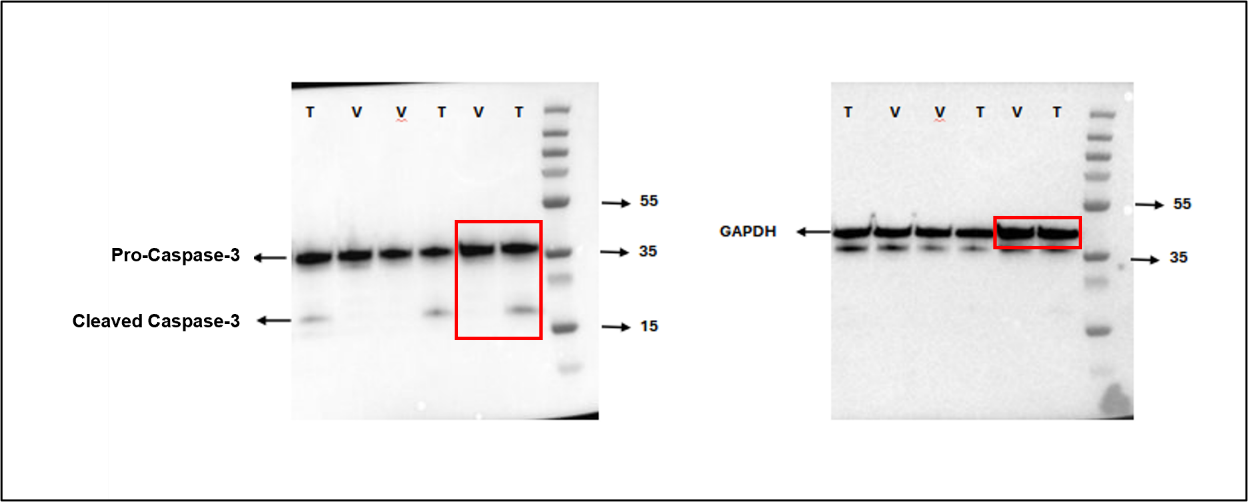
**

**Supplementary Figure S24**. **Standard curve of IL-1β ELISA assay.** The plot shows a 3-degree polynomial fit curve of the IL-1 β ELISA kit standards. The graph was plotted using curve fitting software CurveExpert 1.4.

**Supplementary Figure S25**. **Whole** **Western blot images of CB_1_R.** The images show original Western blot bands of CB_1_R in control cells (C), vehicle treated cells (V) and cisplatin treated cells (T) with respective GAPDH housekeeping.


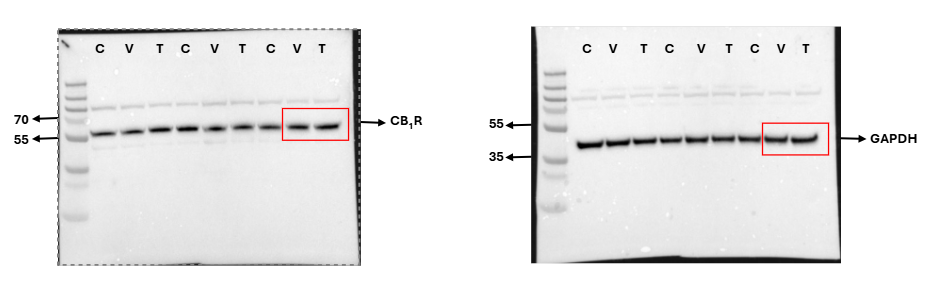


**Supplementary Figure S26**. **Whole** **Western blot images of CB_2_R.** The images show original Western blot bands of CB_2_R in control cells (C) vehicle treated cells (V) and cisplatin treated cells (T) with respective GAPDH housekeeping.


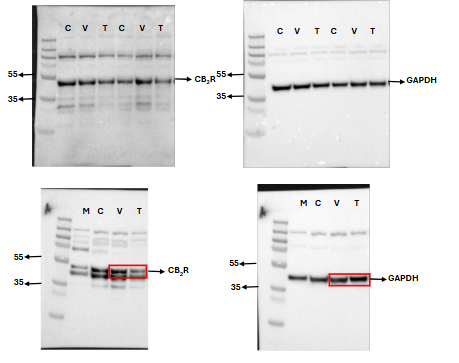


**Supplementary Figure S27**. **Whole** **Western blot images of TRPV 1.** The images show original Western blot bands of TRPV1 in control cells (C), vehicle treated cells (V) and cisplatin treated cells (T) with respective GAPDH housekeeping.


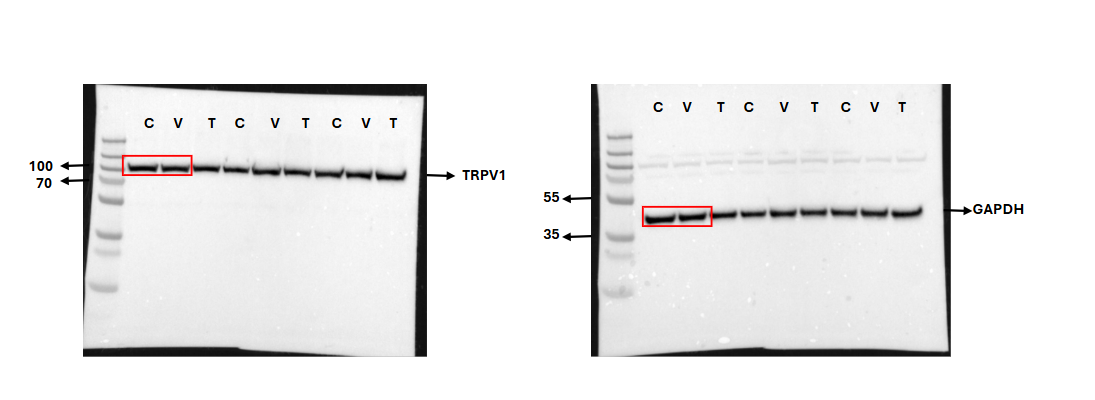


**Supplementary Figure S28.** **Whole** **Western blot images of PPAR α.** The images show original Western blot bands of PPAR α in vehicle treated cells (V) and cisplatin treated cells (T) with respective GAPDH housekeeping.


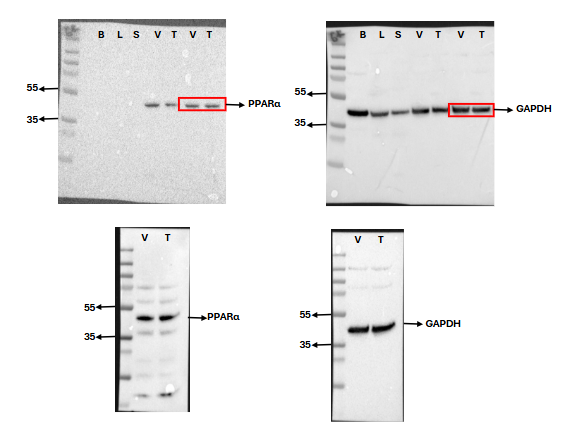


**Supplementary Figure S29**. **Whole** **Western blot images of PPAR δ.** The images show original Western blot bands of PPAR δ in control cells (C), vehicle treated cells (V) and cisplatin treated cells (T) with respective GAPDH housekeeping.


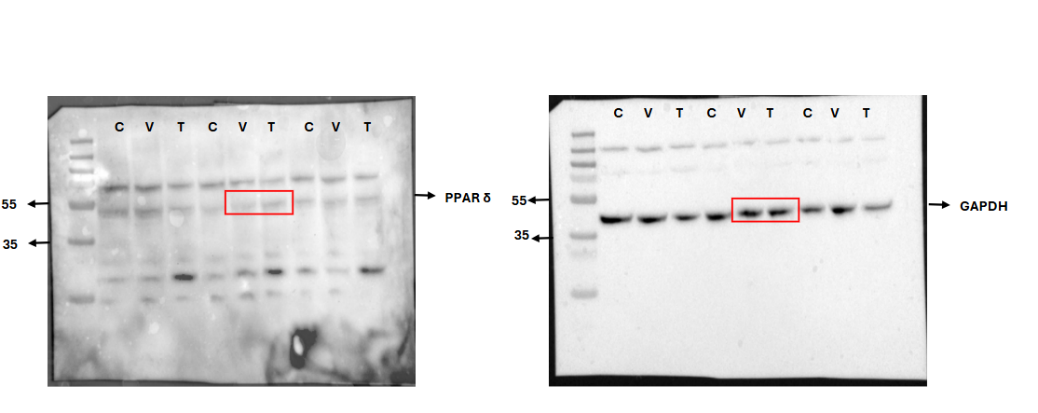


**Supplementary Figure S30**. **Whole** **Western blot images of PPAR γ.** The images show original Western blot bands of PPAR γ in vehicle treated cells (V) and cisplatin treated cells (T) with respective GAPDH housekeeping.


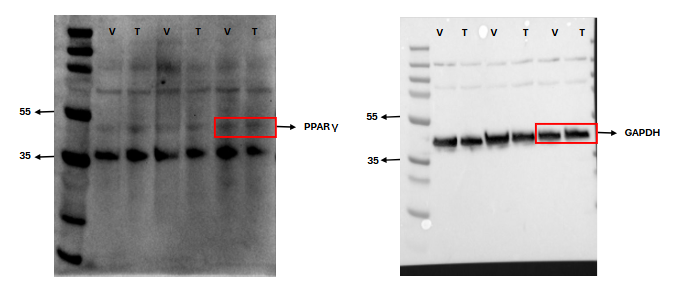


**Supplementary Figure S31**. **Whole** **Western blot images of NAPE-PLD.** The images show original Western blot bands of NAPE-PLD in vehicle treated cells (V) and cisplatin treated cells (T) with respective GAPDH housekeeping. Mouse Brain (B), Liver (L) and Spleen (S) were used as control tissues.


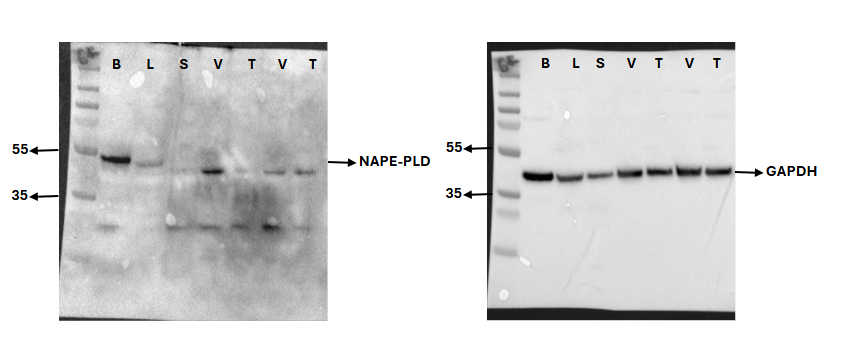


**Supplementary Figure S32.** **Whole** **Western blot images of ABHD 4.** The images show original Western blot bands of ABHD 4 in vehicle treated cells (V) and cisplatin treated cells (T) with respective GAPDH housekeeping.


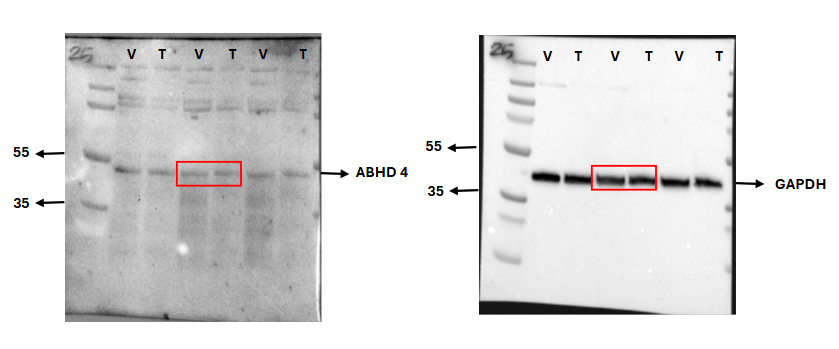


**Supplementary Figure S33.** **Whole** **Western blot images of FAAH.** The images show original Western blot bands of FAAH in vehicle treated cells (V) and cisplatin treated cells (T) with respective GAPDH housekeeping.


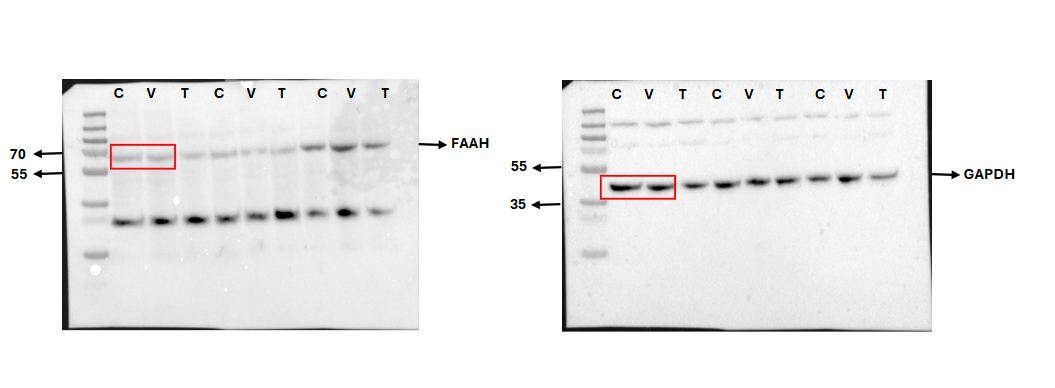


**Supplementary Figure S34**. **Whole** **Western blot images of NAAA.** The images show original Western blot bands of NAAA in vehicle treated cells (V) and cisplatin treated cells (T) with respective GAPDH housekeeping.


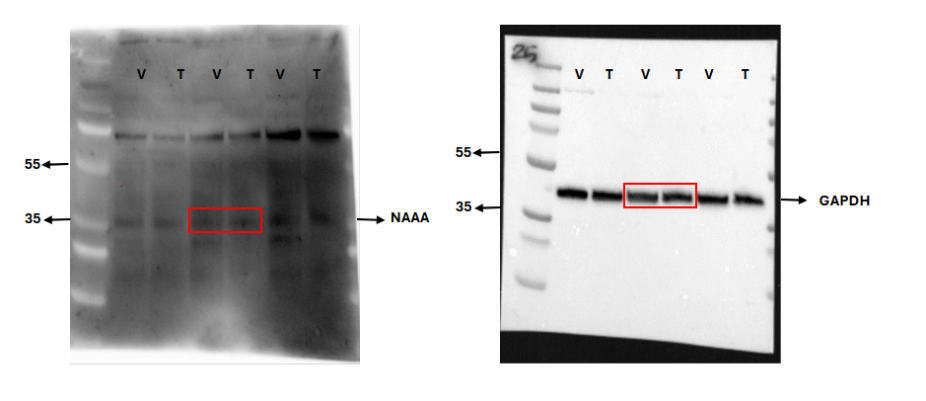


**Supplementary Figure S35.** **Whole** **Western blot images of DAGL β.** The images show original Western blot bands of DAGL β in vehicle treated cells (V) and cisplatin treated cells (T) with respective GAPDH housekeeping.


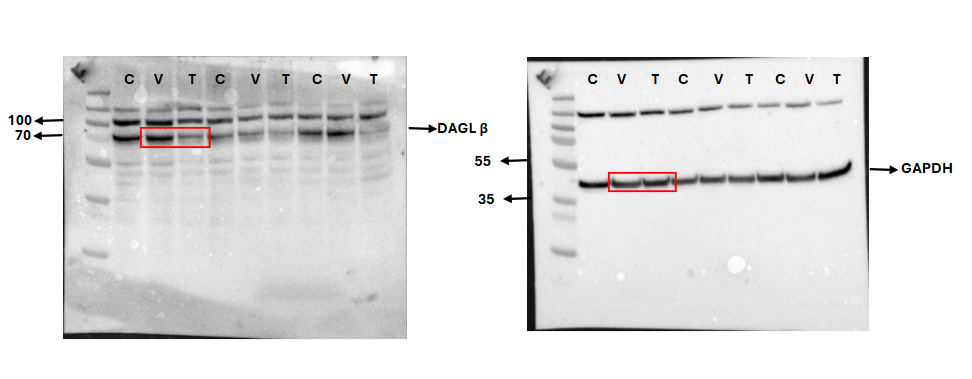


**Supplementary Figure S36.** **Whole** **Western blot images of DAGL α.** The images show original Western blot bands of DAGL α in vehicle treated cells (V) and cisplatin treated cells (T) with respective GAPDH housekeeping. Mouse Brain (B), Liver (L) and Spleen (S) were used as control tissues.


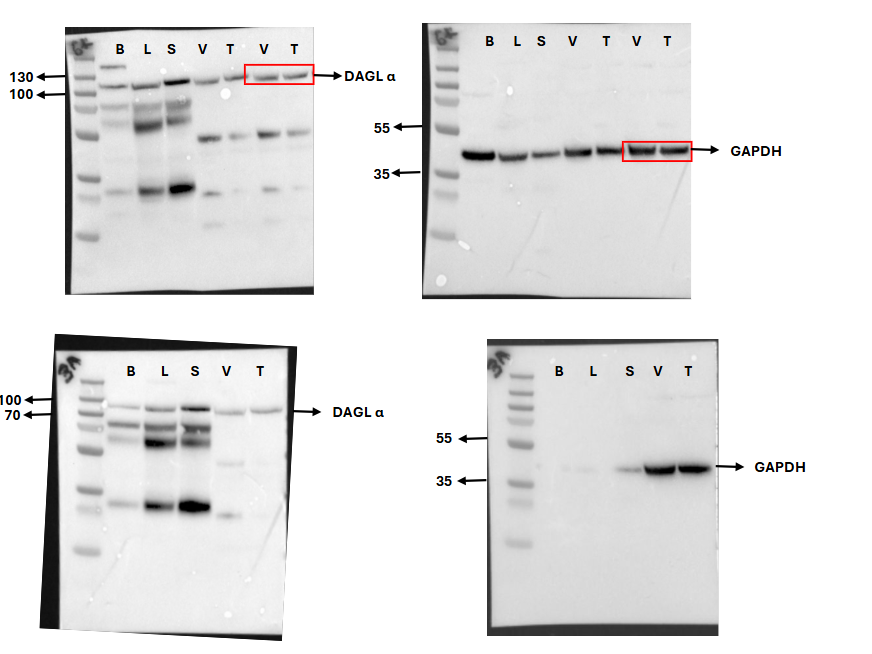


**Supplementary Figure S37. Whole Western blot images of MAGL.** The images show original Western blot bands of MAGL in control cells (C), vehicle treated cells (V) and cisplatin treated cells (T) with respective GAPDH housekeeping.


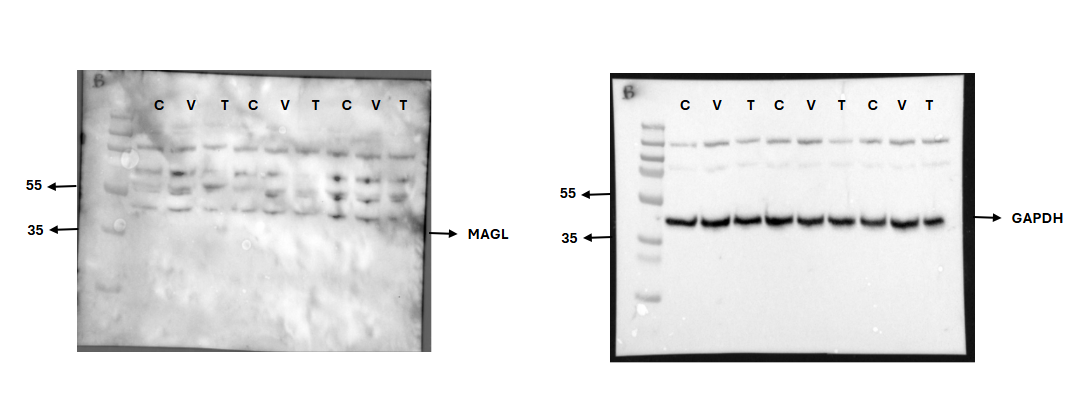


**Supplementary Figure S38.** **Whole** **Western blot images of ABHD 6.** The images show original Western blot bands of ABHD 6 in vehicle treated cells (V) and cisplatin treated cells (T) with respective GAPDH housekeeping. Mouse Brain (B), Liver (L) and Spleen (S) were used as control tissues.


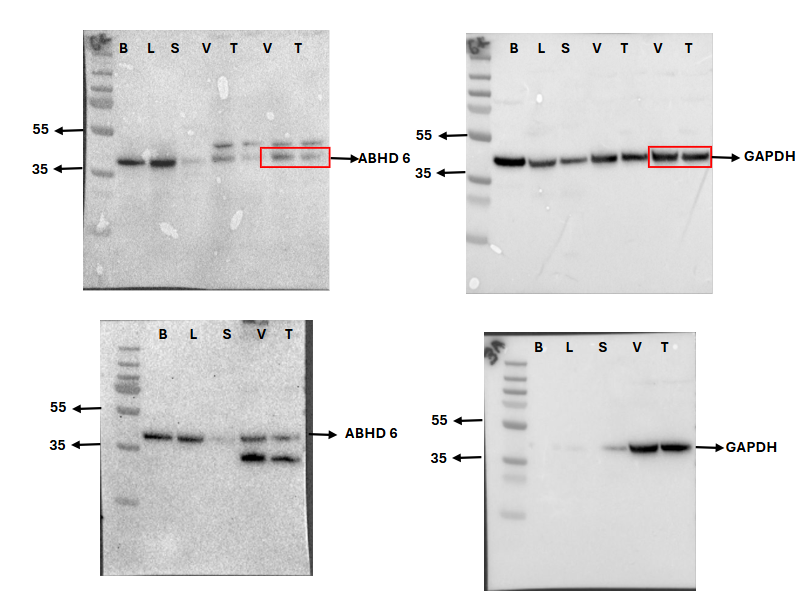


**Supplementary Figure S39.** **Whole** **Western blot images of ABHD 12.** The images show original Western blot bands of ABHD 12 in vehicle treated cells (V) and cisplatin treated cells (T) with respective GAPDH housekeeping.


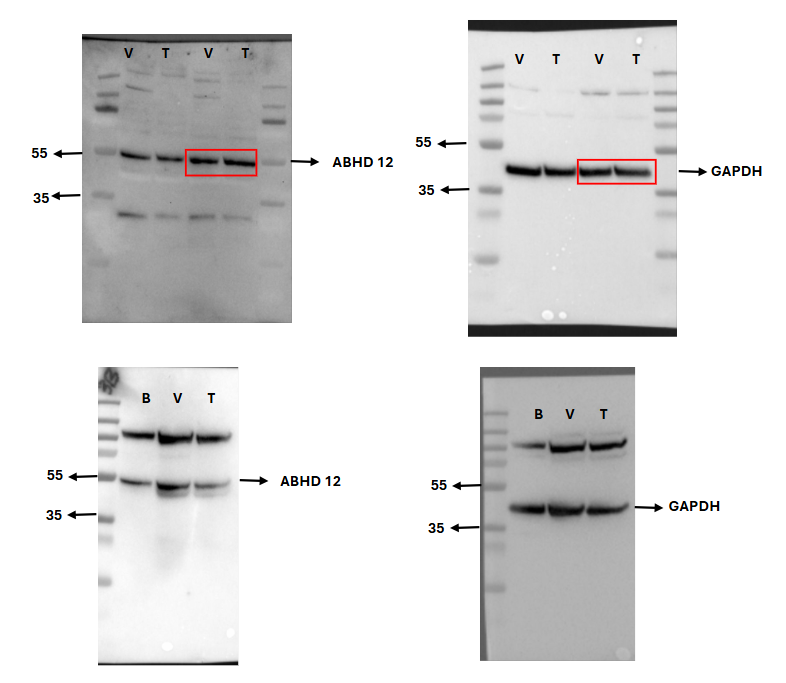


**Supplementary Figure S40. Whole** **Western blot images of Cleaved Caspase-3.** The images show original Western blot bands of Cleaved Caspase-3 in control cells (1), cisplatin treated cells (2) and cisplatin + SR144528 treated cells (3) with respective GAPDH housekeeping.


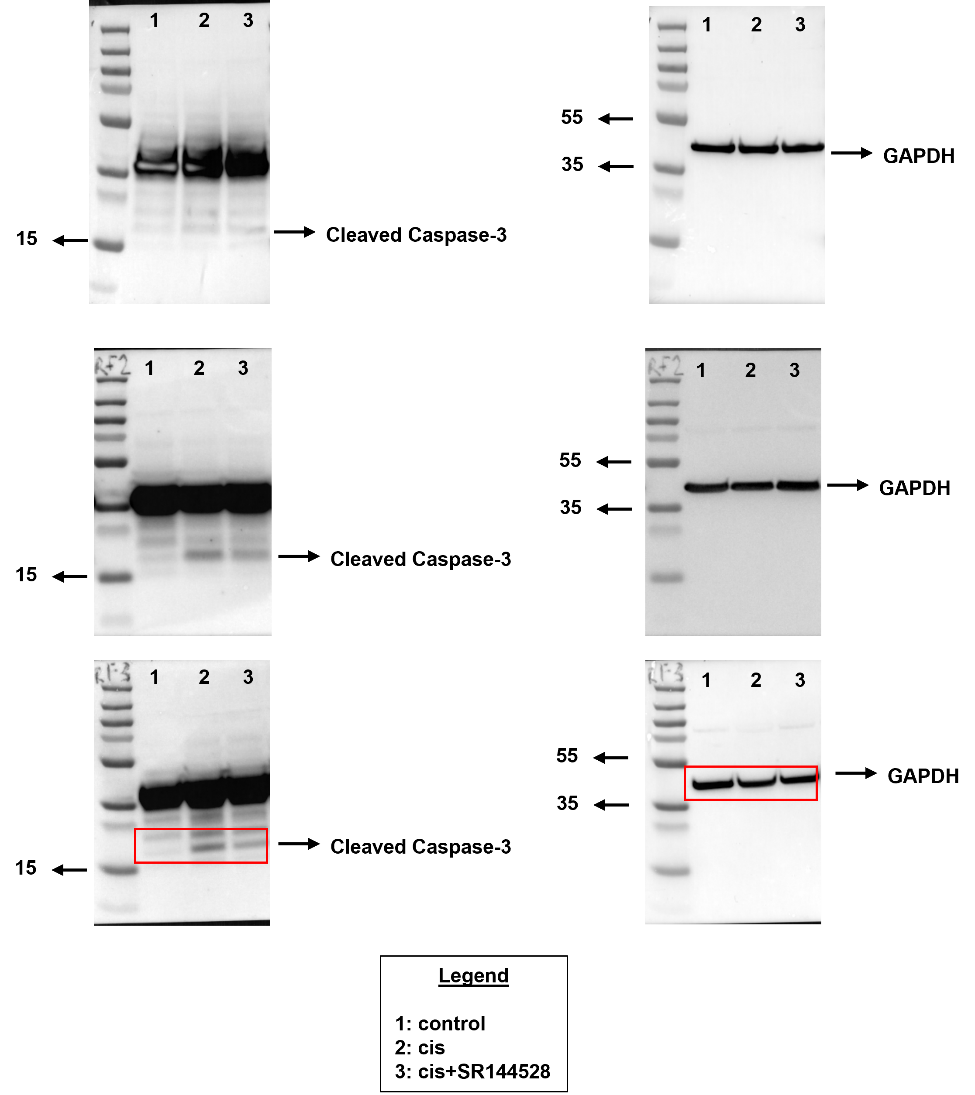


**Supplementary Figure S41. Weights of cisplatin-treated animals.** Weights of each animal (each color represents an animal) measured across the experiment from day 0 to day 7.


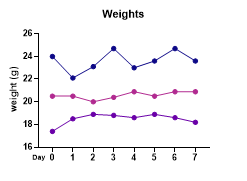


**Supplementary Figure S42. Negative controls for IHC**. Cochlear cryosections incubated with 488-anti-rabbit secondary antibody 1:300 and acquired using the parameters set for anti-CB_2_R immunostaining (A,B); cochlear cryosections incubated with 488-anti-rabbit secondary antibody 1:500 and acquired using the parameters set for anti-DAGLβ and ABHD6 immunostainings (C,D). The sections were counterstained with Hoechst nuclear dye (blue); scale bar-50μm.

**
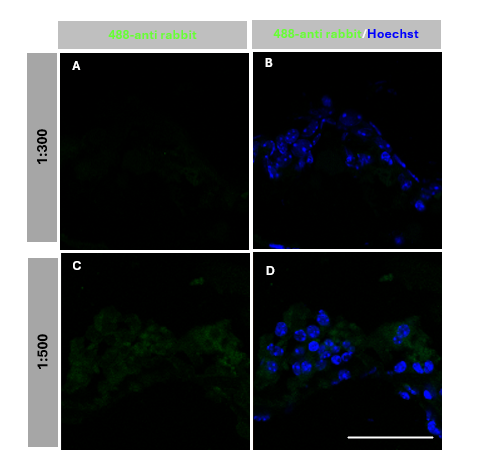
**

**Supplementary Figure S43. Effects of SR144528 treatment on cell viability of UB/OC1 cells.** The graph shows cell viability after treatment with SR144528 (2 μM) on UB/OC1 cells. Data is represented as mean±SEM for 3 independent experiments.


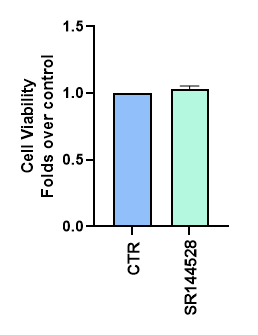


**Supplementary Figure S44. Ratio of biosynthetic-to-degradative enzymes (DAGL β/ABHD 6) in vehicle and cisplatin-treated UB/OC1 cells.** The plot represents the ratio of the protein expression of biosynthetic-to-degradative enzymes (DAGL β/ABHD 6) vehicle (VEH) and cisplatin (CIS)-treated UB/OC1 cells. Data is represented as mean±SEM for 3 independent experiments.


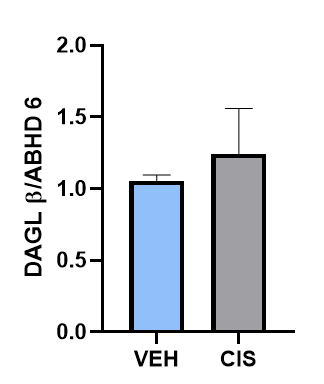


**Supplementary Table S1. eCBs-binding receptors in UB/OC1 cells and in their respective positive controls**

| **RECEPTORS** | **POSITIVE CONTROL** | **REFERENCE FOR THE POSTIVE CONTROL** | **EXPRESSED IN THE CELL LINE (YES/NO)** |
| --- | --- | --- | --- |
| CB_1_R | BRAIN | (67) | YES |
| CB_2_R | LIVER | (68) | YES |
| TRPV1 | BRAIN | (69) | YES |
| PPARα | HEART | (70) | YES |
| PPAR δ | BRAIN | (71) | YES |
| PPAR γ | HEART | (70) | YES |

**Supplementary Table S2: eCBs metabolic enzymes in UB/OC1 cells and in their respective positive controls**

| **ENZYMES** | **POSITIVE CONTROLS** | **REFERENCE FOR THE POSTIVE CONTROL** | **EXPRESSED IN THE UB/OC1 CELL LINE (YES/NO)** |
| --- | --- | --- | --- |
| NAPE-PLD | BRAIN | (72) | NO |
| ABHD4 | BRAIN | (73,74) | YES |
| FAAH | BRAIN | (75-77) | YES |
| NAAA | BRAIN | (78,79) | YES |
| DAGL α | BRAIN | (80-83) | YES |
| DAGL β | BRAIN | (81-83) | YES |
| MAGL | BRAIN | (84,85) | NO |
| ABHD6 | BRAIN | (85,86) | YES |
| ABHD12 | BRAIN | (85) | YES |

**References**

67. Herkenham, M., Lynn, A. B., Little, M. D., Johnson, M. R., Melvin, L. S., De Costa, B. R., and Rice, K. C. (1990) Cannabinoid receptor localization in brain. *Proc Natl Acad Sci U S A* 87, 1932–1936

68. Chen, D. J., Gao, M., Gao, F. F., Su, Q. X., and Wu, J. (2017) Brain cannabinoid receptor 2: Expression, function and modulation. *Acta Pharmacol Sin* 38, 312–316

69. Menigoz, A. and Boudes, M. (2011) The expression pattern of TRPV1 in brain. *Journal of Neuroscience* 31, 13025–13027

70. Lee, W. S. and Kim, J. (2015) Peroxisome proliferator-activated receptors and the heart: Lessons from the past and future directions. *PPAR Res* 2015, 271983

71. Warden, A., Truitt, J., Merriman, M., Ponomareva, O., Jameson, K., Ferguson, L. B., Mayfield, R. D., and Harris, R. A. (2016) Localization of PPAR isotypes in the adult mouse and human brain. *Sci Rep* 6, 27618

72. Egertová, M., Simon, G. M., Cravatt, B. F., and Elphick, M. R. (2008) Localization of N-acyl phosphatidylethanolamine phospholipase D (NAPE-PLD) expression in mouse brain: A new perspective on N- acylethanolamines as neural signaling molecules. *Journal of Comparative Neurology* 506, 604–615

73. Lee, H. C., Simon, G. M., and Cravatt, B. F. (2015) ABHD4 regulates multiple classes of N -acyl phospholipids in the mammalian central nervous system. *Biochemistry* 54, 2539–2549

74. Lord, C. C., Thomas, G., and Brown, J. M. (2013) Mammalian alpha beta hydrolase domain (ABHD) proteins: lipid metabolizing enzymes at the interface of cell signaling and energy metabolism. *Biochim Biophys Acta* 1831, 792

75. Fowler, C. J., Jonsson, K. O., and Tiger, G. (2001) Fatty acid amide hydrolase: Biochemistry, pharmacology, and therapeutic possibilities for an enzyme hydrolyzing anandamide, 2-arachidonoylglycerol, palmitoylethanolamide, and oleamide. *Biochem Pharmacol* 62, 517–526

76. Cravatt, B. F., Saghatelian, A., Hawkins, E. G., Clement, A. B., Bracey, M. H., and Lichtman, A. H. (2004) Functional disassociation of the central and peripheral fatty acid amide signaling systems. *Proc Natl Acad Sci U S A* 101, 10821–10826

77. Pathak, S., Kumar, K. R., Kanta, H., Carr-Johnson, F., Han, J., Bashmakov, A., Faure, L., Ding, H., Vanarsa, K., Khan, S., Li, Q.-Z., Chapman, K., Wakeland, E. K., and Mohan, C. (2016) Fatty Acid Amide Hydrolase Regulates Peripheral B Cell Receptor Revision, Polyreactivity, and B1 Cells in Lupus. *The Journal of Immunology* 196, 1507–1516

78. Tai, T., Tsuboi, K., Uyama, T., Masuda, K., Cravatt, B. F., Houchi, H., and Ueda, N. (2012) Endogenous molecules stimulating N-acylethanolamine-hydrolyzing acid amidase (NAAA). *ACS Chem Neurosci* 3, 379–385

79. Xie, X., Li, Y., Xu, S., Zhou, P., Yang, L., Xu, Y., Qiu, Y., Yang, Y., and Li, Y. (2022) Genetic Blockade of NAAA Cell-specifically Regulates Fatty Acid Ethanolamides (FAEs) Metabolism and Inflammatory Responses. *Front Pharmacol* 12, 817603

80. Schurman, L. D., Carper, M. C., Moncayo, L. V., Ogasawara, D., Richardson, K., Yu, L., Liu, X., Poklis, J. L., Liu, Q. S., Cravatt, B. F., and Lichtman, A. H. (2019) Diacylglycerol lipase-alpha regulates hippocampal-dependent learning and memory processes in mice. *Journal of Neuroscience* 39, 5949–5965

81. Viader, A., Ogasawara, D., Joslyn, C. M., Sanchez-Alavez, M., Mori, S., Nguyen, W., Conti, B., and Cravatt, B. F. (2016) A chemical proteomic atlas of brain serine hydrolases identifies cell type-specific pathways regulating neuroinflammation. *Elife* 5, e12345

82. Reisenberg, M., Singh, P. K., Williams, G., and Doherty, P. (2012) The diacylglycerol lipases: Structure, regulation and roles in and beyond endocannabinoid signalling. *Philosophical Transactions of the Royal Society B: Biological Sciences* 367, 3264–3275

83. Baggelaar, M. P., Chameau, P. J. P., Kantae, V., Hummel, J., Hsu, K. L., Janssen, F., Van Der Wel, T., Soethoudt, M., Deng, H., Den Dulk, H., Allarà, M., Florea, B. I., Di Marzo, V., Wadman, W. J., Kruse, C. G., Overkleeft, H. S., Hankemeier, T., Werkman, T. R., Cravatt, B. F., and Van Der Stelt, M. (2015) Highly Selective, Reversible Inhibitor Identified by Comparative Chemoproteomics Modulates Diacylglycerol Lipase Activity in Neurons. *J Am Chem Soc* 137, 8851–8857

84. Pasquarelli, N., Porazik, C., Hanselmann, J., Weydt, P., Ferger, B., and Witting, A. (2015) Comparative biochemical characterization of the monoacylglycerol lipase inhibitor KML29 in brain, spinal cord, liver, spleen, fat and muscle tissue. *Neuropharmacology* 91, 148–156

85. Blankman, J. L., Simon, G. M., and Cravatt, B. F. (2007) A Comprehensive Profile of Brain Enzymes that Hydrolyze the Endocannabinoid 2-Arachidonoylglycerol. *Chem Biol* 14, 1347–1356

86. Thomas, G., Betters, J. L., Lord, C. C., Brown, A. L., Marshall, S., Ferguson, D., Sawyer, J., Davis, M. A., Melchior, J. T., Blume, L. C., Howlett, A. C., Ivanova, P. T., Milne, S. B., Myers, D. S., Mrak, I., Leber, V., Heier, C., Taschler, U., Blankman, J. L., Cravatt, B. F., Lee, R. G., Crooke, R. M., Graham, M. J., Zimmermann, R., Brown, H. A., and Brown, J. M. (2013) The serine hydrolase ABHD6 Is a critical regulator of the metabolic syndrome. *Cell Rep* 5, 508–520

1. 2-AG: 2-Arachidonoylglycerol; AA: Arachidonic acid; ABHD 4/6/12: α/β Hydrolase Domain-Containing Protein; AEA: *N*-arachidonoylethanolamine;  ASC: Apoptosis-associated speck-like protein; BSA: Bovine Serum Albumin; CB_1_R: cannabinoid receptor 1; CB_2_R: cannabinoid receptor 2; CIS: cisplatin; COX: Cyclooxygenase; DAG: Diacylglycerol; DAGL α/β: Diacylglycerol lipases α and β; DHEA: docosahexaenoylethanolamine; DMSO: Dimethyl sulfoxide; ECS: Endocannabinoid system; eCBs: Endocannabinoids; ELISA: Enzyme linked immunosorbent assay; EPEA: epoxyeicosatetraenoyl ethanolamide; EtNH_2_: Ethanolamine; FAAH: Fatty acid amide hydrolase; FDA: Food and Drug administration; GAPDH: Glyceraldehyde-3-Phosphate Dehydrogenase; GPCR: G-protein coupled receptor; GSDMD: Gasdermin-D; LOX: Lipoxygenase; HC: Hair cells; IC_50_: Half-maximal inhibitory concentration; IHC: Immunohistochemistry; IP_3_R: Inositol-3-phosphate receptor; LEA: Linoleoylethanolamide; LPS: lysophosphatidylserine; MAGL: Monoacylglycerol lipase; MTT: 3-(4,5-dimethylthiazol-2-yl)-2,5-diphenyltetrazolium bromide; Myo7a: Myosin 7a; NAAA *N*-acylethanolamine-hydrolyzing acid amidase; NAPE-PLD *N*-acylethanolamines-specific phospholipase D; NarPE: *N*-arachidonoyl-phosphatidylethanolamine; NF-κB: Nuclear factor kappa-light-chain-enhancer of activated B-cells; NH: Normal hearing; NLRP3: pyrin domain–containing-3; OEA: *N*-oleoylethanolamine; OC: organ of Corti; ; PBS: Phosphate Buffered Saline; PEA: *N*-palmitoylethanolaminePHARC: polyneuropathy, hearing loss, ataxia, retinosis pigmentosa, and cataract; POEA: Palmitoleoyl Ethanolamide; PPAR α, γ, δ: ; pe SPL: peak equivalent sound pressure level; Peroxisome proliferator-activated nuclear receptors α, γ, δ; PUFA: polyunsaturated fattyacid; RT: Room temperature; SEA: *N*-stearoylethanolamine; SNHL: sensorineural hearing loss; TRPV1: Transient receptor potential vanilloid receptor 1; VEH: vehicle. [↑](#footnote-ref-1)
